# Let-7g suppresses both canonical and non-canonical NF-κB pathways in macrophages leading to anti-atherosclerosis

**DOI:** 10.18632/oncotarget.18197

**Published:** 2017-05-23

**Authors:** Yung-Song Wang, Edward Hsi, Hsin-Yun Cheng, Shih-Hsien Hsu, Yi-Chu Liao, Suh-Hang H. Juo

**Affiliations:** ^1^ Institute of Fisheries Science, National Taiwan University, Taipei, Taiwan; ^2^ Department of Life Science, National Taiwan University, Taipei, Taiwan; ^3^ Department of Medical Research, China Medical University Hospital, Taichung, Taiwan; ^4^ Graduate Institute of Medicine, Kaohsiung Medical University, Kaohsiung, Taiwan; ^5^ Department of Neurology, National Yang-Ming University School of Medicine, Taipei, Taiwan; ^6^ Department of Neurology, Taipei Veterans General Hospital, Taipei, Taiwan; ^7^ Graduate Institute of Biomedical Sciences, China Medical University, Taichung, Taiwan; ^8^ Institute of New Drug Development, China Medical University, Taichung, Taiwan; ^9^ Brain Disease Research Center, China Medical University Hospital, Taichung, Taiwan

**Keywords:** atherosclerosis, foam cell, let-7, microRNA, macrophage

## Abstract

Transformation of macrophages to foam cells contributes to atherosclerosis. Here, we report that let-7g reduces macrophage transformation and alleviates foam cell apoptosis by suppressing both canonical and non-canonical NF-κB pathways. In the canonical pathway, let-7g inhibits phosphorylation of IKKβ and IκB, down-regulates SREBF2 and miR-33a, and up-regulates ABCA1. In the non-canonical pathway, let-7g directly knocks down MEKK1, IKKα and ablates IKKα phosphorylation. Let-7g's effects in macrophages can be almost completely blocked by inactivation of NF-κB signaling, which suggests that let-7g's effects are primarily mediated through the suppression of NF-κB pathways. NF-κB has been reported to directly activate lin28 transcription, and lin28 is a well-known negative regulator for let-7 biogenesis. Therefore, there is negative feedback between NF-κB and let-7g. Additional macrophages-specific NF-κB knockout in the apoE deficiency mice reduces atherosclerotic lesion by 85%. Let-7g also suppresses p53-dependent apoptosis. Altogether, sufficient let-7g levels are important to prevent NF-κB over-activation in macrophages and to prevent atherosclerosis.

## INTRODUCTION

Macrophages play a critical role in host responses of the innate immune system. Dysregulation of macrophage function is involved in many pathological conditions, including atherosclerosis and inflammatory diseases [1]. Uptake of modified lipids, in particular, oxidized low-density lipoprotein (oxLDL) by macrophages causes their transformation into lipid-laden foam cells [2], which leads to atherosclerotic changes and can eventually result in overt cardiovascular diseases [3]. There are continuous efforts to search for solutions to prevent foam cell formation.

NF-κB transcription factors are pivotal regulators of inflammation and cell death in the pathogenesis of atherosclerosis [4]. NF-κB activation is tightly controlled by a number of endogenous mechanisms that limit excessive and prolonged production of pro-inflammatory mediators. p105 and p100 are two mammalian NF-κB proteins and they mediate the stimulus-caused signaling pathway to activate IκB kinase (IKK). The IKK complex contains two catalytic subunits, IKKα and IKKβ, and a regulatory subunit IKKγ. IKKβ can be phosphorylated and activated by kinases that have not been fully clarified, while IKKα is phosphorylated by NF-κB-inducing kinase (NIK) [5]. In the canonical NF-κB signaling pathway, the activation of IKK complex (IKKα, IKKβ and IKKγ) causes IκBα/IκBb phosphorylation and subsequent ubiquitin-dependent IκB degradation by the proteasome complex [6, 7]. This event leads to nuclear translocation of the NF-κB RelA/p50 heterodimers and then activation of NF-κB downstream genes. In the NF-κB non-canonical signaling pathway, the homodimers of IKKα directly phosphorylate p100 resulting in subsequent degradation to p52 by the proteasome [8, 9], which allows p52 to activate transcription of downstream genes.

MicroRNAs (miRNAs) are important regulators of gene expression [10, 11], and they are non-coding, single-stranded RNA molecules of about 21–23 nucleotides in length. The annealing of the miRNA to its target mRNA causes an inhibition of protein translation, and/or cleavage of the mRNA. Our group recently reported that miRNA let-7g can bind to the 3′-untranslated region (3′UTR) of LOX-1 mRNA to prevent the entry of oxLDL into blood vessel [12], and let-7g improves endothelial functions by directly inhibiting the TGF-β signaling pathway [13]. Given that macrophages play important roles in the inflammatory response and lipid deposition in the artery, this study aimed to elucidate the role of let-7g in the NF-κB pathway in macrophages in the context of atherosclerosis.

## RESULTS

### Let-7g inhibits macrophage transformation to a foam cell

**Figure 1:**
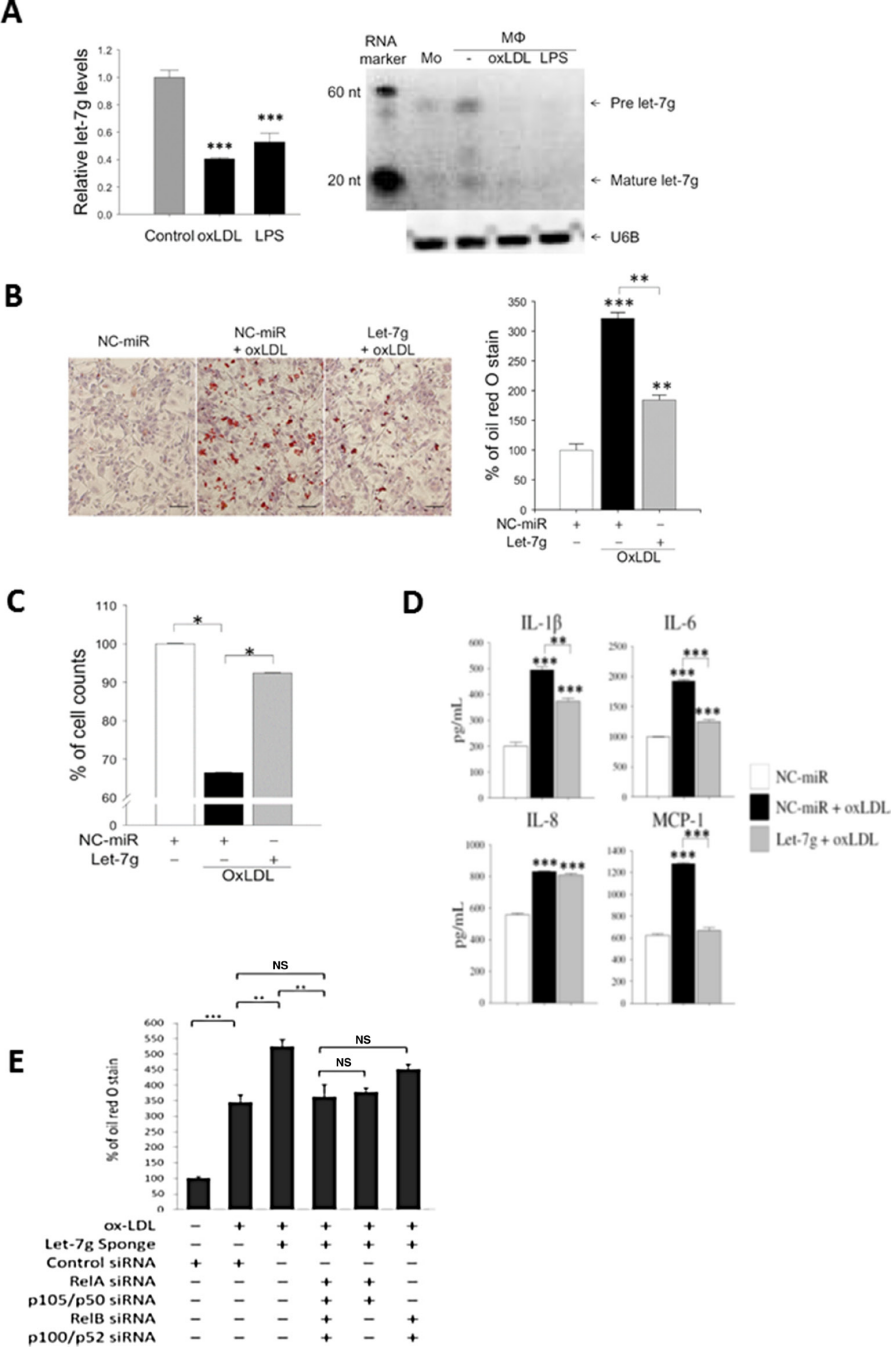
Let-7g reduces foam cell formation, increases macrophage viability and decreases inflammation Macrophages were differentiated from THP-1 cells. (**A**) let-7g RNA expression levels in macrophages treated with oxLDL (40 μg/ml) or LPS (100 ng/ml), respectively, for 24 hours. (**B**) Macrophages were transfected with let-7g mimic (50 nmol/L) for 24h, and then stained with oil red O (ORO). Scale bar, 100 μm. The quantified data were shown in the bar chart. (**C**) Cell viability was determined by trypan blue stain at 24 h after microRNA transfection. (**D**) Cytokine and chemokine in the culture medium of let-7g-transfected and oxLDL-treated macrophages. The data were measured by ELISA. (**E**) Knockdown of let-7g by LV-let-7g-sponge caused a significant increase of lipid accumulation, but the effect was reversed by inhibiting canonical signaling (5th bar), non-canonical signaling (6th bar) or both signaling pathways (4th bar) by siRNAs for NF-κB transcription factors. The data were from at least three independent experiments. Data in each bar chart are presented as mean ± SEM ^*^P < 0.05, ^**^ *P* < 0.01, ^***^P < 0.001.

THP-1 cells are pre-monocytes and can be differentiated into macrophage-like cells. During the induction of THP-1 to macrophages, let-7g expression was increased by 1.3-fold (*P* < 0.01). When macrophages were further transformed to foam cells by the stimulation of oxLDL (40 μg/ml) or lipopolysaccharide (LPS, 100 ng/ml), let-7g expression was reduced by more than 50% (*p* < 0.001 for either oxLDL or LPS treatment) (Figure 1A). The quantitative data on let-7g northern blot were shown in Supplementary Figure 1. These results implied that let-7g may be involved in foam cell formation.

oxLDL treatment caused lipid accumulation and cell death in macrophages. Transfecting let-7g mimic (50 nmol/L) to oxLDL-treated macrophages resulted in a decrease of lipid accumulation (Figure 1B) and an increase of cell count (Figure 1C). In addition, let-7g mimic significantly reduced the secretion of pro-inflammatory substances including IL-1β, IL-6 and MCP-1 (all *P* < 0.001; Figure 1D), but had no effect on IL-8. On the contrary, transfection of let-7g sponge vector significantly increased lipid accumulation in the oxLDL-treated macrophages (Figure 3rd bar vs 2nd bar Figure 1E).

**Figure 2:**
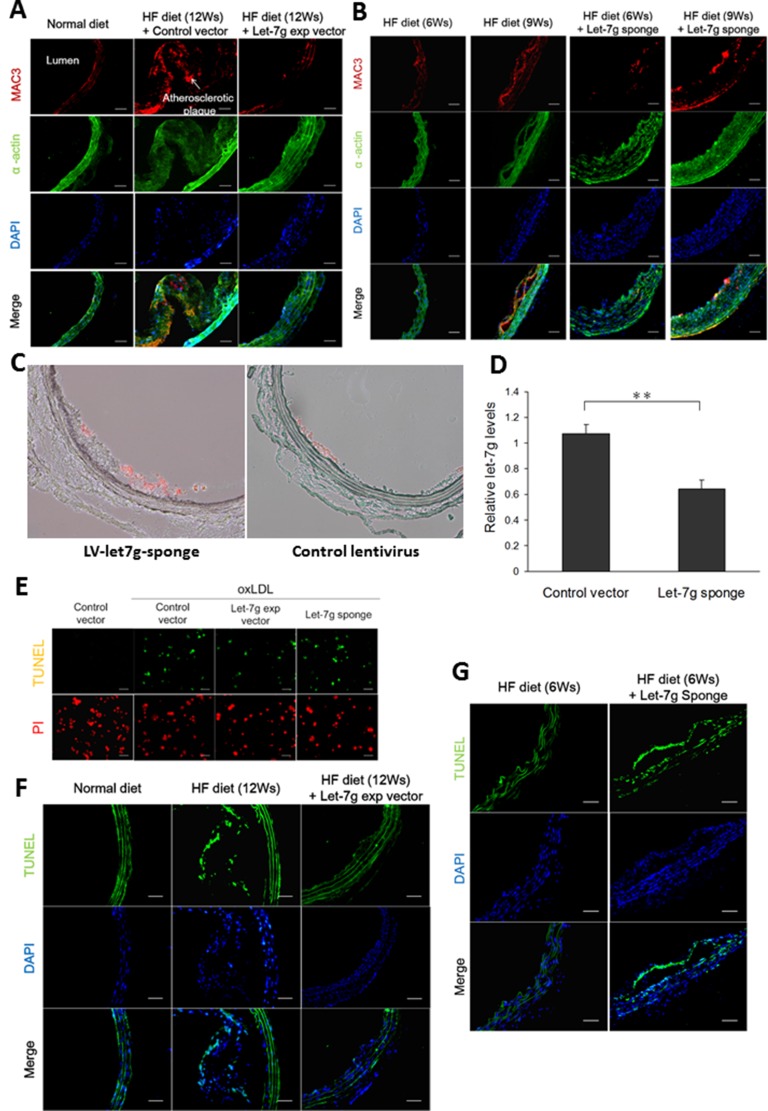
Let-7g decreases macrophage accumulation in the plaques, reduces the plaque size and exerts anti-apoptotic effect (**A**) Atherosclerotic plaques developed in the aortas of apoE KO mice (*n* = 6 per group) under a HF diet for 12 weeks, but the plaque sizes were substantially reduced when the animals were injected with LV-let7g. Macrophages (red) and VSMC (green) were indicated by the MAC3 and α-actin immunfluorescent staining, respectively. Scale bar, 50 μm. (**B**) The atherosclerotic plaques of apoE KO mice (*n* = 6 per group) injected with LV-let7g-sponge under a HF diet for 6 or 9 weeks. Scale bar, 50 μm. (**C**, **D**) Representative aortic slices from apoE KO mice treated with LV-let7g-sponge (left) or control lentivirus (right) for 9 weeks while under a HF diet. Macrophages (red) were indicated by the MAC3. let-7g level in the macrophages dissected from the atherosclerotic plaques by laser capture micro-dissection was quantified in the bar chart. To measure let-7g levels, RNU6B was used as the internal control. (**E**) The TUNEL assay for macrophages infected with LV-let7g, LV-let7g-sponge or LV-control vector. Scale bar, 100μm. (**F**, **G**) TUNEL-positive cells (green) in aortic sections of apoE KO mice (*n* = 6 per group) treated with LV-let7g or LV-let7g-sponge. Scale bars, 50 μm. The data were from at least three independent experiments. Data in each bar chart are presented as mean ± S.E.M. ^*^P < 0.05, ^**^P < 0.01, ^***^P < 0.001.

To confirm the protective effect of let-7g *in vivo*, lentivirus carrying let-7g expressing vector (LV-let7g) was used in the animal study. We first infected lentivirus carrying let-7g expressing vector (LV-let7g) 250 MOI to macrophages, which increased let-7g expression by 8.8-fold compared with the control lentivirus. Then apoE KO mice were intravenously injected with LV-let7g weekly for 12 weeks, which led to substantial reduction of atherosclerotic plaques in the aorta (Figure 2A). MAC3 is an antigen expressed on macrophages and can be used to identify the existence of macrophages in the blood vessel. We found that let-7g reduced macrophage accumulation in the arterial wall of apoE KO mice (Figure 2A, and Supplementary Figure 2 for quantitative data) leading to an anti-atherosclerotic effect, but let-7g had less effect on suppressing the proliferation of vascular smooth muscle cells (VSMC, indicated by α-actin; Figure 2A). Conversely, lentivirus carrying let-7g sponge (LV-let7g-sponge) was constructed to knock down endogenous let-7g by more than 50% in macrophages. Injection of LV-let7g-sponge to apoE KO mice accelerated macrophage aggregation after 6- to 9-week treatment while the mice were under a high-fat diet (Figure 2B and Supplementary Figure 3 for quantitative data). To further confirm that the injected LV-let7g-sponge could decrease let-7g levels in macrophages, we used laser capture microdissection to obtain MAC3 positive cells from aortas of apoE KO mice treated by LV-let7g-sponge for 9 weeks (Figure 2C). The results showed that LV-let7g-sponge significantly (*P* < 0.01) reduced vascular macrophage let-7g levels by almost 50% than the control lentivirus (Figure 2D). Accordingly, LV-let7g-sponge exerts a similar effect on macrophage let-7g in both *in vitro* and *in vivo* experiments. Taken together, these *in vitro* and *in vivo* results suggested that let-7g plays an important role to reduce macrophage transformation into a foam cell and suppress the arterial plaque formation.

The increased macrophage cell count by let-7g (Figure 1C) could be mediated by anti-apoptosis or cell proliferation. Using the TUNEL assay, we showed that let-7g exerted anti-apoptosis *in vitro* (Figure 2E and Supplementary Figure 4 for quantitative data) and *in vivo* (the aortas of apoE KO mice under a HF diet, Figure 2F and Supplementary Figure 5 for quantitative data). On the contrary, LV-let7g-sponge increased cell apoptosis in both cellular (Figure 2E and Supplementary Figure 4 for quantitative data) and animal studies (Figure 2G and Supplementary Figure 6 for quantitative data).

### Let-7g suppresses P53-depedent apoptosis in macrophages

**Figure 3:**
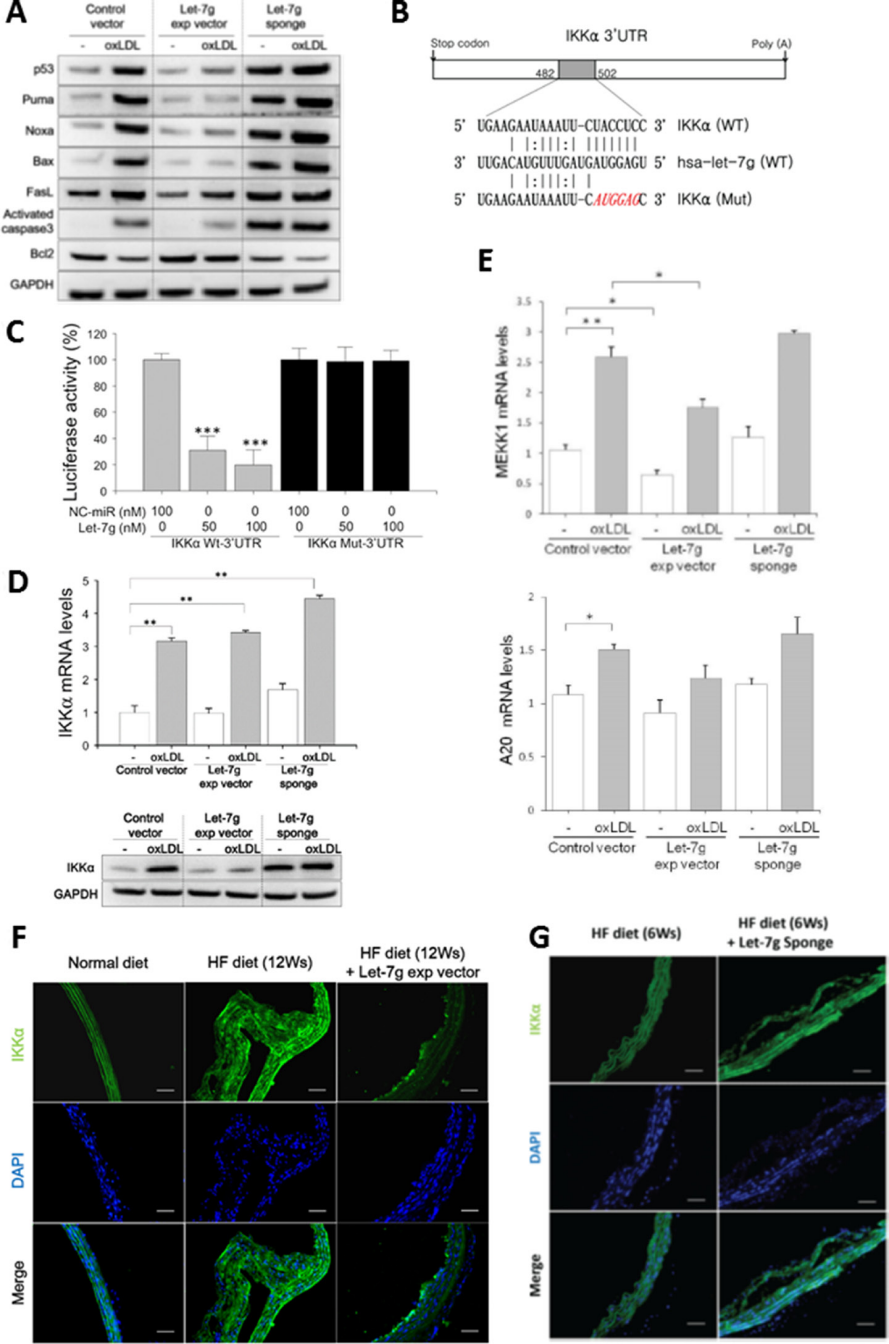
let-7g suppresses NF-κB signaling leading to anti-apoptosis for macrophages (**A**) let-7g affected gene expression in the p53-signaling in oxLDL-treated macrophages. (**B**) Bioinformatic predicted let-7g's binding site in the 3′-UTR of the IKKα transcript. (**C)** let-7g dose-dependently knocked down luciferase activity in the cells carrying wild-type IKKα plasmid, but let-7g had no effect on luciferase activity in the cells carrying the mutant IKKα plasmid. (**D**) let-7g knocked down IKKα expression in macrophages. (**E**) Let-7g's effects on mRNA levels of MEKK1 and A20 in macrophages. (**F**, **G**) IKKα was detected by the immunofluorescent staining in apoE KO mice (*n* = 6 per group) under a HF diet for 6 or 12 weeks. LV-let7g treatment reduced the plaque size and IKKα expression *in vivo*, and LV-let7g-sponge had an opposite effects. Scale bars: 50 μm. Data are presented as mean ± SEM from 3 independent experiments. ^*^ : < 0.05; ^**^ : < 0.01; ^***^ : < 0.001.

We further tested whether let-7g anti-apoptotic effect is via p53 pathway. Our experiment showed that LV-let7g suppressed p53-dependent apoptotic pathway by down-regulating p53, Puma, Noxa, Bax and Caspase-3 protein levels but LV-let7g did not have a significant effect on FasL (Figure 3A and Supplementary Figure 7 for quantitative data). On the contrary, these apoptotic proteins were up-regulated and anti-apoptotic Bcl2 was down-regulated by LV-let7g-sponge (Figure 3A). The results implied that let-7g can reduce macrophage (or foam cell) apoptosis via suppression of p53-dependent apoptotic pathway.

### Let-7g suppresses NF-κB signaling in macrophages

Macrophages play a critical role in regulating inflammation. The NF-κB family of transcription factors has an essential role in inflammation and innate immunity. Therefore, we speculated that let-7g may also affect NF-κB pathway in macrophages. To search for let-7g target genes in the NF-κB signaling pathway, we first used the ingenuity pathway analysis (IPA, http://www.ingenuity.com/products/ipa) to identify 29 NF-κB related genes (Supplementary Figure 8 and Supplementary Table 2). Among these 29 genes, three genes (IKKα, MEKK1 and A20) were strongly predicted as let-7g target genes by the TargetScan database, and IKKα is most related to the NF-κB signaling. We used the luciferase reporter assay to confirm that let-7g could directly bind to IKKα 3′UTR (Figure 3B and 3C). IKKα protein levels were reduced by let-7g as shown by western blot (Figure 3D and Supplementary Figure 9 for quantitative data), although let-7g did not alter IKKα mRNA level (Figure 3D). Infecting LV-let7g to macrophages significantly reduced MEKK1 but not A20 mRNA levels (Figure 3E).

Consistent with cellular studies, LV-let7g treatment substantially decreased the IKKα protein in the aortas of apoE KO mice under a 12-week HF diet (Figure 3F and Supplementary Figure 10 for quantitative data). As expected, LV-let-7g-sponge treatment up-regulated the IKKα expression in the aortas of apoE KO mice even in just 6-week treatment (Figure 3G and Supplementary Figure 11 for quantitative data). The above findings confirmed that let-7g could affect several genes involved in the NF-κB pathway.

### Let-7g inhibits both canonical and non-canonical NF-κB signaling

**Figure 4:**
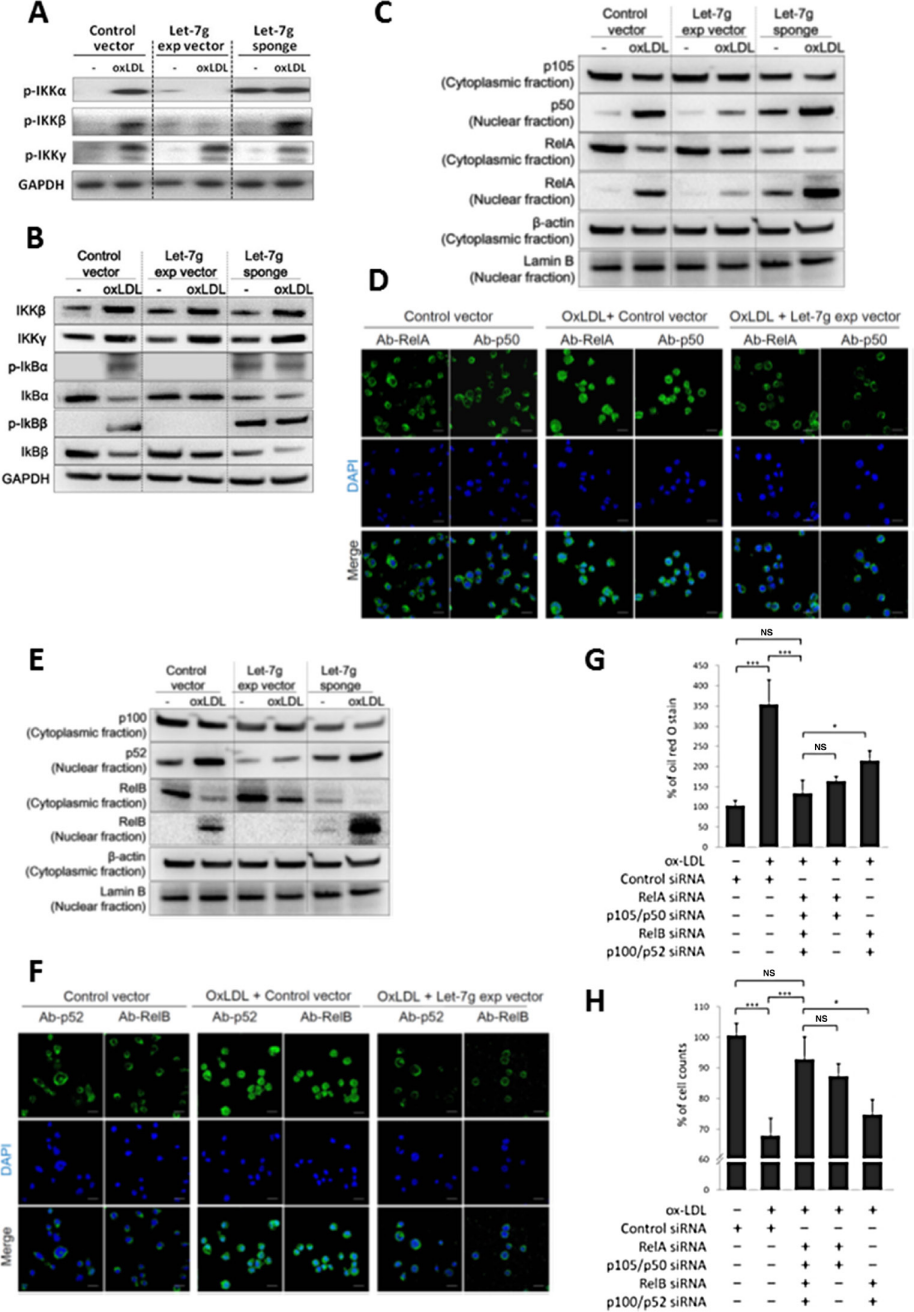
Let-7g inhibits canonical and non-canonical NF-κB signaling pathways Macrophages were differentiated from THP-1 cells and were infected with LV-let7g, LV-let7g-sponge or control lentivirus. (**A**) Western blot analysis for phosphorylated- IKKα, phosphorylated-IKKβ and phosphorylated-IKKγ. GAPDH was served as a loading control. (**B**) Western blot analysis for IKKβ, IKKγ, IκBα, IκBβ, phosphorylated-IκBα (p-IκBα) and p-IκBβ. GAPDH was served as a loading control. (**C**) Cellular distribution of transcription factors in the canonical pathway. p105, p50 and RelA in the cytoplasm and nucleus with cytoplasmic β-actin and nuclear lamin B as the respective markers. (**D**) The confocal laser-scanning microscopy revealed the localization of fluorescently tagged p50 and RelA. Coloring indicated GFP (green) and the DNA dye DAPI (blue). Scale bar: 20 μm. (**E**) Cellular distribution of transcription factors in the non-canonical pathway. p100, p52 and RelB proteins in the cytoplasm and nucleus with cytoplasmic β-actin and nuclear lamin B as the respective markers. (**F**) The confocal laser-scanning microscopy showed the localization of GFP-tagged p52 and RelB proteins. Scale bar: 20 μm. (**G**, **H**). Knockdown of the canonical components (RelA and p105, 5th bar), or non-canonical components (RelB and p52, 4th bar) caused different effects on (G) intracellular lipid accumulation and (H) cell counts in macrophages. The data were from at least three independent experiments. Data in each bar chart are presented as mean ± SEM ^*^P < 0.05, ^**^P < 0.01, ^***^P < 0.001.

In addition to direct suppression of the translation of IKKα gene, let-7g also suppresses the signal transduction of the IKK upstream genes (such as MEKK1). Phosphorylation of IKK subunits is essential to activate IKK complex and NF-κB downstream pathway. Based on our data on Western blot, oxLDL could promote NF-κB signal transduction in macrophages (Figure 4A). However, the oxLDL effects on NF-κB signaling could be suppressed by let-7g because let-7g totally blocked IKKα phosphorylation, substantially decreased IKKβ phosphorylation but had no effect on IKKγ in oxLDL-treated macrophages (Figure 4A and Supplementary Figure 12 for quantitative data). The results indicated that let-7g not only repressed the IKKα-dependent non-canonical pathway, but also inhibited the canonical pathway where IKKα was considered to be dispensable. Therefore, we further explored let-7g's effects on each individual NF-κB pathway.

Let-7g over-expression completely suppressed the downstream IκBα and IκBβ phosphorylation and partially decreased IκB degradation (Figure 4B and Supplementary Figure 13 for quantitative data). Subsequently, we confirmed that let-7g attenuated the nuclear translocation of RelA/p50 dimer in oxLDL-treated macrophages by western blot (Figure 4C and Supplementary Figure 14 for quantitative data) and immunofluorescence confocal microscope (Figure 4D and Supplementary Figure 15 for quantitative data), which suggested let-7g could inhibit canonical signaling. Although let-7g could not totally suppress IKKα expression (Figure 3D), it almost completely ablated IKKα phosphorylation (Figure 4A and Supplementary Figure 12 for quantitative data). Let-7g significantly decreased nuclear translocation of p52, and almost completely inhibited RelB nuclear translocation in oxLDL-treated macrophages (Figure 4E and 4F, quantitative data were shown in Supplementary Figures 16 and 17, respectively), which suggested let-7g could inhibit non-canonical signaling.

To test for the effect of each NF-κB signaling transduction on macrophages, we first knocked down the canonical signaling by RelA-siRNA and p105/p50-siRNA. The data showed that oxLDL had mild and non-significant effect on lipid accumulation and cell apoptosis if the NF-κB canonical pathway is blocked (Figure 4G and 4H). On the contrary, when the non-canonical signaling was inhabited by RelB-siRNA and p100/p52-siRNA, oxLDL still caused significant lipid accumulation and cell apoptosis (Figure 4G and 4H). Such data indicated that the canonical signaling plays a major role in foam cell formation and apoptosis. We further compared the let-7g effect with NF-κB effect on lipid accumulation in macrophages. In our cell model, removal of let-7g increased lipid accumulation (3rd bar in Figure 1E), but this effect was completely reversed by total inactivation of NF-κB signaling (4th bar in Figure 1E). Accordingly, the results suggested that let-7g's effect on intracellular lipid accumulation is almost entirely mediated through the suppression of NF-κB signaling.

### Let-7g influences the downstream genes of canonical and non-canonical pathways

**Figure 5:**
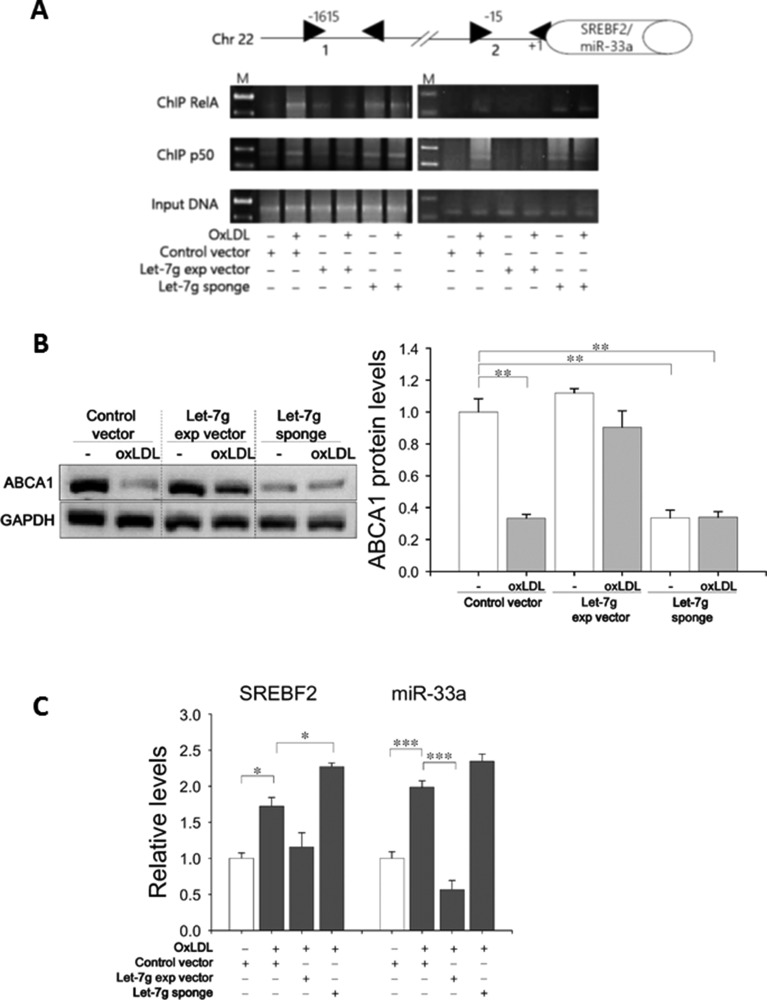
Let-7g increases ABCA1 expression by suppressing miR-33a (**A**) 2 putative RelA/p50-binding sites in the SREBF2 promoter. Macrophages infected with LV-let7g-sponge or LV-let7g were used for the ChIP assay by using RelA/p50 antibodies or an irrelevant antibody (Ig, nonimmune IgG). M: molecular weight marker (100 bp ladders). (**B**) Western blot analysis of ABCA1 protein in macrophages. (**C**) SREBF2 mRNA and miR-33a levels in macrophages. Data are presented as mean ± SEM from 3 independent experiments. ^*^P < 0.05; ^**^P < 0.01; ^***^P < 0.001.

We further investigated the canonical pathway-dependent lipid accumulation. ABCA1 is a major molecule to export excess cholesterol in macrophages to reduce foam cell formation [14]. ABCA1 can be induced via the NF-κB canonical pathway [15] and can be down-regulated by miR-33 [16]. The miR-33a gene is in the intron of the SREBF2 gene, and SREBF2 is a transcription factor that plays an important role in cholesterol biosynthesis. Accordingly, the relationship among let-7g, ABCA1, SREBF2/miR-33a and NF-κB was examined. First, two NF-κB binding sites (at -1615 and -15 bp) in the SREBF2 promoter were predicted by the TFSEARCH (www.cbrc.jp/research/db/TFSEARCH.html) (Figure 5A). ABCA1 protein levels were significantly decreased by oxLDL, but let-7g could reverse ABCA1 expression (Figure 5B). Furthermore, SREBF2 and miR-33a expression levels were decreased by LV-let7g and increased by LV-let7g-sponge (Figure 5C). The ChIP assay confirmed that let-7g could decrease oxLDL-stimulated RelA and p50 binding to the SREBF2 promoter (Figure 5A), but LV-let7g-sponge had an opposite effect (Figure 5A). The above results indicated that let-7g enhanced ABCA1-mediated cholesterol efflux by inhibiting the NF-κB canonical pathway that elevates SREBF2/miR-33a expression.

We used the ChIP-sequencing (ChIP-Seq) and RNA deep sequencing (RNA-seq) to identify the genes altered by the non-canonical signaling during the transformation of macrophages into foam cells. First, 768 highly confident p52-binding sites were discovered according to p52-ChIP-seq when macrophages were treated with oxLDL and the canonical pathway was inhibited (Supplementary Figure 18). The RNA-seq discovered a total of 412 differentially expressed genes caused by RelB/p52 (defined as P < 0.05 and fold change > 2). The combined results from the ChIP-seq and RNA-seq revealed 12 genes that are substantially and directly affected by p52 in the non-canonical pathway. Annotation of these 12 genes with gene ontology (GO) terms showed that they are involved in macrophage movement, foam cell formation, response to oxidative stress and cell apoptosis (Supplementary Table 3).

### Macrophage-specific NF-κB KO reduces atherosclerosis in mice

**Figure 6:**
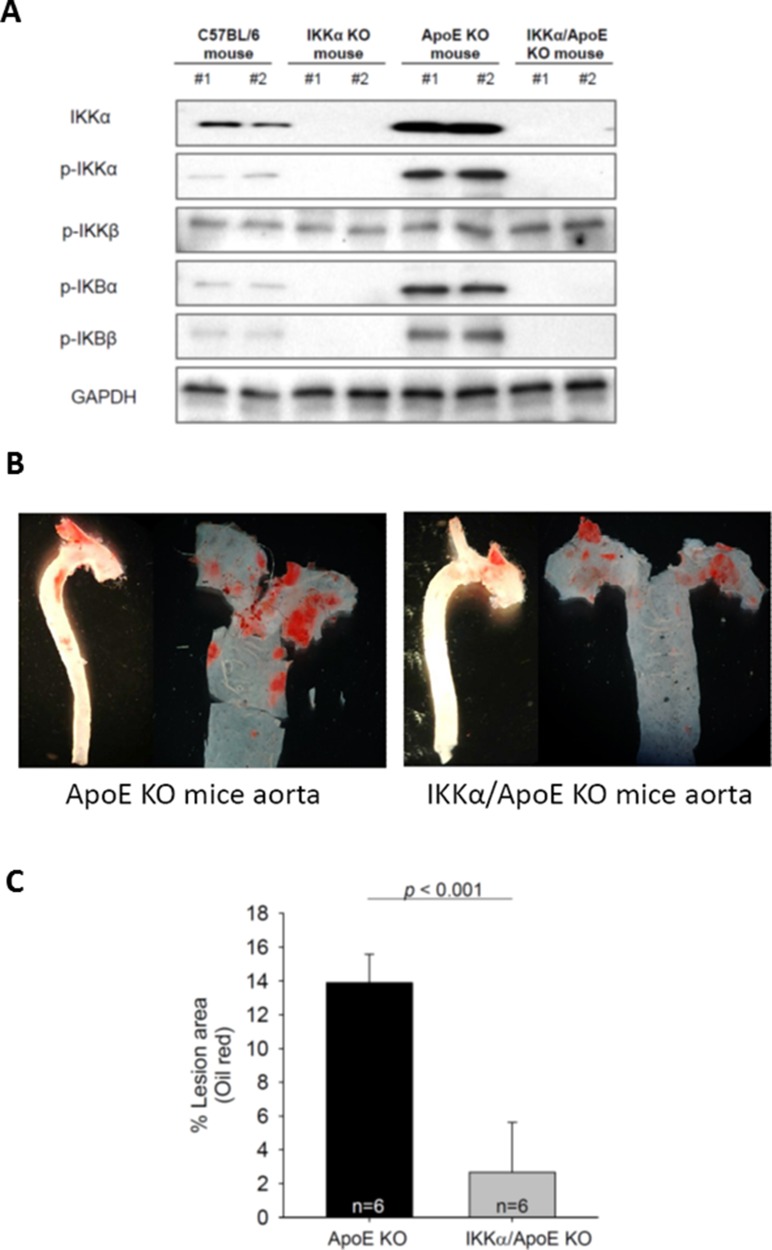
Macrophage-specific NF-κB KO in the apoE KO (IKKαf/f:MLysCre/apoE-/-) mice significantly diminishes atherosclerosis (**A**) NF-κB related proteins in macrophages of different types of mice. (**B**) Representative pictures of atherosclerotic plaques in the aortas of apoE KO and IKKαf/f:MLysCre/apoE^-/-^ mice under a 12-week HF diet. (**C**) The percentage of aorta occupied by the atherosclerotic plaques (*n* = 6 per group). The data were from at least three independent experiments. Data in each bar chart are presented as mean ± SEM ^*^P < 0.05, ^**^P < 0.01, ^***^P < 0.001.

Macrophage-specific IKKα KO (IKKα*^f/f^*:MLysCre) mice had no NF-κB activity in macrophages (Figure 6A). Among 4 different types of mice (normal C57BL/6, IKKα*^f/f^*:MLysCre, apoE KO, and IKKα*^f/f^*:MLysCre and apoE double KO (IKKα*^f/f^*:MLysCre/apoE^-/-^) mice), there was no activation of IκBs and no detectable IKKα protein in macrophages from IKKα*^f/f^*:MLysCre and IKKα*^f/f^*:MLysCre/apoE^-/-^ mice (Figure 6A), while p-IκBs and IKKα protein were highly expressed in macrophages from apoE KO and C57BL/6 mice. The data on NF-κB related proteins in macrophages from IKKα*^f/f^*:MLysCre mice (Figure 6A) was similar to the data on let-7g treated macrophages derived from THP-1 cells (Figure 4A and 4B). Since the IKKα*^f/f^*:MLysCre/apoE^-/-^ mouse is incapable to activate either canonical or non-canonical pathway, it can be used to test macrophage-specific let-7g effects on atherosclerosis. The lesion of atherosclerotic plaques in the aortas were compared between IKKα*^f/f^*:MLysCre/apoE^-/-^ and apoE KO mice under a HF diet for 12 weeks. The data showed that the IKKα*^f/f^*:MLysCre/apoE^-/-^ double knockout mice had a reduction of atherosclerotic lesions by 85% (p < 0.001) than apoE KO mice based on quantification en face analysis (Figure 6B and 6C). Such data confirmed that significant atherosclerosis formation can be attributed to NF-κB activity in macrophages.

## DISCUSSION

**Figure 7:**
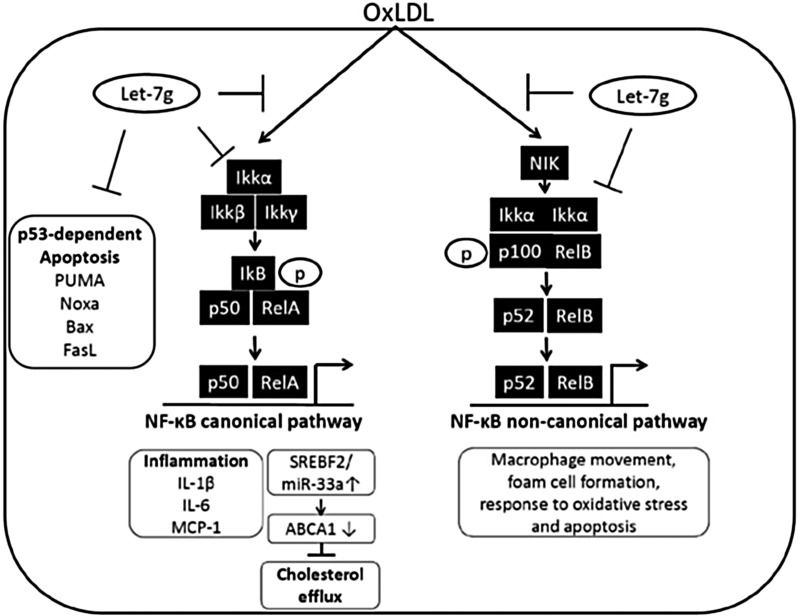
Schematic illustration for let-7g's influence on NF-κB signaling in macrophages In macrophages, let-7g can target NF-κB complex and its upstream molecules to knock down both canonical and non-canonical pathways. Through inhibition of the canonical pathway in macrophages, let-7g can reduce cell apoptosis, decrease inflammation and promote cholesterol efflux. The inhibition of non-canonical pathway leads to suppress macrophage movement, foam cell formation, response to oxidative stress and cell apoptosis.

The present study shows that let-7g could suppress both NF-κB canonical and non-canonical signaling pathways in macrophages to reduce intracellular lipid accumulation, and secretion of inflammatory substances. Let-7g also reduced macrophage apoptosis via suppression of p53-dependent pathway. In the NF-κB signaling, let-7g exerted inhibitory effects on multiple places including knockdown of IKKα and MEKK1, suppression of phosphorylation of IKKα, IKKβ and decrease of IκB degradation. The IKKα*^f/f^*:MLysCre mouse presented deficiency in canonical and non-canonical activation in macrophages, which is similar to let-7g effects on the NF-κB signaling. Consistently, the IKKα*^f/f^*:MLysCre/apoE^-/-^ mice had much milder atherosclerosis than apoE KO mice. Furthermore, the treatment of LV-let7g-sponge to apoE KO mice not only reduced let-7g levels in macrophages, but also increased both atherosclerosis and macrophage/foam cell apoptosis. These *in vivo* data further implied the importance of let-7g in preventing atherosclerosis by regulating cholesterol homeostasis and reducing inflammation in macrophages. This is the first time to illustrate the detailed mechanism of let-7g in regulating the NF-κB pathway. Our findings are schematically summarized in Figure 7.

Our previous study has shown a HF diet can suppress let-7g expression [12], while a HF diet was reported to increase IKKα expression in animals [17]. It has been reported that NF-κB can directly activate Lin28 transcription, and Lin28 is a well-known negative regulator for let-7 biogenesis [18]. Accordingly, there is reciprocally negative feedback between let-7g and NF-κB.

oxLDL caused an increase of lipid accumulation by 3.5-fold (1st vs 2nd bar in Figure 4G), but knock-down of NF-κB restricted the oxLDL effect to approximately 1.3-fold (1st vs 3rd bar in Figure 4G). When the NF-κB signaling was intact and let-7g level was suppressed, oxLDL caused the highest lipid accumulation in macrophages by 5-fold (i.e. 3rd bar in Figure 1E). A decrease of let-7g significantly increased lipid accumulation in macrophages by 43% (2^nd^ bar vs 3^rd^ bar in Figure 1E). However, such an increase was almost totally reversed by knocking down NF-κB signaling using siRNAs (i.e. 3rd bar vs 4th bar in Figure 1E). The data suggest that maintaining a sufficient level of let-7g is important to prevent NF-κB over-activation. Furthermore, our results indicate that oxLDL-induced lipid accumulation is primarily due to activation of NF-κB signaling.

Let-7g can regulate cholesterol metabolism in macrophages by inhibiting RelA/p50 nuclear translation. In the nucleus, RelA/p50 can up-regulate SREBF2 and miR-33a levels resulting in a low level of ABCA1 expression. Notably, ABCA1 plays a critical role in exporting cholesterol from macrophages to protect against the development of atherosclerosis. In addition, recent studies have shown that SREBF2 is a critical regulator of cholesterol/lipid homeostasis by controlling the expression of many cholesterogenic and lipogenic genes [16]. miR-33a has been implied to elevate cholesterol accumulation in macrophages, and suggested to be related to inflammatory response, cell cycle, cell proliferation, and glucose homeostasis [19]. Although previous studies have reported that let-7g may suppress the NF-κB canonical pathway [20], the detailed mechanism on how let-7g affects the NF-κB canonical pathway has not been not identified.

The NF-κB canonical pathway is known for its role in atherosclerosis, while the involvement of the NF-κB non-canonical pathway is less studied. IKKα, but not IKKβ or IKKγ, is required for NF-κB non-canonical signaling [21]. The activated IKKα dimer phosphorylates and degrades p100 to free its N-terminal proportion (p52) that enters the nucleus together with RelB. Because let-7g not only represses IKKα expression but also blocks IKKα phosphorylation, p100 processing can be decreased and the nuclear translocation of RelB/p52 is subsequently reduced. Based on the data shown in Figure 4G and 4H, activation of non-canonical pathway alone (i.e. 4th bar in both Figures) still had mild detrimental effects on lipid accumulation and cell viability. We specifically investigated which genes can be affected by RelB/p52 in oxLDL-treated macrophages, and 12 genes affected by the non-canonical signaling were identified. Further investigation of these genes may provide more insight to the physiological function of non-canonical pathway.

The IKK complex is essentially made of two kinases (IKKα and IKKβ) and a regulatory subunit, IKKγ. We showed that let-7g could directly knock down IKKα and totally ablate IKKα phosphorylation, which blocked the non-canonical cascade. Let-7g also blocked IKKβ phosphorylation and therefore the inactivated IKKβ did not have the kinase effect on IkB leading to a failure of RelA/p50 translocation to the nucleus. Although IKKα has been considered dispensable in activating the canonical pathway in several cell types [22, 23], our data on IKKαf/f:MLysCre mice showed that IKKα may not be dispensable for IkB activation for the canonical pathway in macrophages. The data from our IKKαf/f:MLysCre/apoE^-/-^ animal study were comparable with the results from a previous animal study using endothelial cell-restricted inhibition of canonical pathway [24]. On the other hand, a recent study reported that mice developed significant atherosclerotic changes when inactivatable IKKα^AA/AA^ variant exists in the bone marrow cells [25]. Their study showed that RelA activation was not different between IKKα^AA/AA^ and IKKα^+/+^ in macrophages [25], which indicated the canonical pathway was active in IKKα^AA/AA^ macrophages. In contrast, macrophages from our IKKαf/f:MLysCre/apoE^-/-^ mice had no activation in canonical or non-canonical pathway. Combining data from our and Tilstam et al's studies [25], it is likely that IKKαmay act as an adaptor protein for IkB phosphorylation in macrophages. However, the exact mechanism for IKKα influence on IkB phosphorylation needs further investigation.

We recently showed that let-7g can reduce atherosclerotic change by improving endothelial functions and by reducing oxLDL uptake by VSMC and endothelial cells [12, 13, 26]. Here we demonstrated an additional role of let-7g in macrophages, which further supports the importance of let-7g in maintaining vascular health. Indeed, our previous study has shown that let-7g reduced monocyte adhesion to endothelial cells by suppressing the TGF-ß pathway, which can reduce macrophage formation in the blood vessels [13]. Furthermore, here we showed that let-7g could decrease p53-dependent apoptosis in macrophages, which is consistent with a similar report for let-7g's anti-apoptotic effect on endothelial cells [27]. Therefore, the increased apoptotic cells in Figure 2F and 2G might be mediated through anti-let-7g's effects on both macrophages and endothelial cells.

In conclusion, let-7g can substantially suppress NF-κB activation in macrophages, which causes a reduction of foam cell formation, anti-apoptosis and anti-inflammation in macrophages. The present study augments the potential of using let-7g as the therapeutic agent to treat or prevent atherosclerosis.

## MATERIALS AND METHODS

### THP-1 and macrophage cell culture

Human THP-1 monocytic leukemia cells were obtained from the American Type Culture Collection. THP-1 cells were maintained in RPMI 1640 medium with 10% heat-inactivated FCS, 50 U/mL of penicillin G, and 50 mg/mL of streptomycin sulfate in an atmosphere containing 5% CO_2_ and 95% air. Cultures were maintained at a cell concentration between 2 × 10^5^ and 1 × 10^6^ cells/mL, with culture medium added every 2 days. Treating THP-1 cells with 200 nM phorbol myristate acetate (PMA) for 48 hours can induce differentiation to macrophages. Cells were seeded at a density of 1.5 × 10^5^ cells per well on collagen coated 12-well plates and maintained at 37°C in a 5% CO_2_ atmosphere. Lipopolysaccharide (LPS; 100 ng/ml) were used to induce foam cell formation, inflammatory substances and cell apoptosis. Cell viability was determined by Trypan Blue. Murine macrophages were differentiated from bone marrow progenitors from the femurs of gene**-**knockout mice by M-CSF conditioned DMEM/10% FBS for 7 days [28].

### Oil red O staining

THP-1 derived macrophages were seeded onto multiwell slides at 5 × 10^4^ cells. The cells were washed 3 times with PBS, fixed with formaldehyde, and stained with oil red O (ORO) and hematoxylin. In order to obtain quantitative data, 1 ml of isopropyl alcohol was added to the stained culture dish. After 5 minutes, the absorbance of the extract was assayed by a spectrophotometer at 510 nm after dilution to a linear range.

### Enzyme-linked immunosorbent assay for cytokine levels

THP-1 derived macrophages transfected with let-7g mimic were incubated in the presence or absence of 40 μg/ml oxLDL for 24 h, and the supernatants of conditioned medium were collected for the measure of IL-1β, IL-6, IL-8 and MCP-1 levels by the ELISA kits (BD Bioscience) according to the manufacturer's protocol.

### RNA extraction and real-time PCR

Total RNA was isolated from the cells by Trizol reagent (Invitrogen) according to the manufacturer's instructions. RNA quality was confirmed using the A260/A280 reading. cDNA was synthesized by reverse transcription with 1 μg RNA using a random primer and the SuperScript™ kit (Invitrogen). The 20 μl reverse transcription products were diluted to 100 μl and 2 to 3 μl was used for real-time PCR.

Real-time PCRs were performed in duplicate using 5 μl 2× SYBR Green PCR Master Mix, 0.2 μl primer sets, 1 μl cDNA and 3.6 μl nucleotide-free H_2_O, to yield a 10 μl reaction. All the reactions were amplified on a 7900 HT Fast Real Time PCR system (Life Technologies). For let-7g and RNU6B detection, cDNA was synthesized from TaqMan^®^ MicroRNA assays. Real-time PCR was performed in duplicate to measure let-7g expression (Assay ID:000494) using the specific TaqMan MiRNA Reverse Transcription kit (Life Technologies). For let-7g detection, cDNA was synthesized from TaqMan MicroRNA assays and RNU6B was used as the internal control. Specific primers used in real-time PCR for human IKKα, p53 and GAPDH are listed in Table EV1, and GAPDH are listed in Supplementary Table 1, and GAPDH was used as the reference gene. The expression ratios were calculated as the normalized CT difference between the control and sample with the adjustment for the amplification efficiency relative to the expression level of the reference gene.

### Northern blotting assay for let-7g

For the use of the northern blot assay kit (Signosis, Sunnyvale, CA) for let-7g detection, RNA (5 mg/well) was fractionated using 15% TBE urea-PAGE, blotted on membranes, and hybridized with biotin labeled let-7g and U6 probes. The amount of RNA loaded in each well was normalized to the amount of U6.

### Gene knockout mice

Apolipoprotein E knockout (apoE KO) mice was purchased from the Jackson Laboratory, while conditional IKKα knockout mice were a gift from Dr. Kenneth Marcu (Stony Brook University). Mice with IKKα alleles flanked by LoxP recombination sites (IKKαf/f mice) that express Cre recombinase under the control of the macrophage lysozyme (MLys) promoter in mature macrophages and neutrophils (IKKαf/f:MLysCre mice) have been described previously [29]. Both the apoE KO and IKKαf/f:MLysCre mice are on the C57BL/6 background. IKKαf/f:MLysCre mice were further intercrossed with apoE KO mice to generate IKKαf/f:MLysCre/apoE^-/-^ mice. IKKα^f/f^:MLysCre, and apoE KO mice were screened for Cre and deletion of apoE gene by genotyping as described previously [30], and the primers used in genotyping are listed in Supplementary Table 1.

### Animal experiment

Mice were raised in a controlled environment at 20 ± 2 °C, with 40–70% relative humidity and an artificial 12-h light-dark cycle. Weight gain was monitored every week and food intake twice a day during the period of each study. IKKα*^f/f^*:MLysCre/apoE^-/-^ and apoE KO mice were fed with 4g/day of high fat diet containing 0.2% cholesterol (Testdiet® 57BD) that is known to elicit fatty streak lesions in the arteries of susceptible mice. The Animal Care and Use Committee of the Kaohsiung Medical University approved the animal experimental protocols (IACUC Approval No: 103029), which strictly conformed to the Guide for the Care and Use of Laboratory Animals, 8th Edition (2011).

Lentiviral expression vector pCDH-CMV-MCS-EF1-GreenPuro (SBI Mountain View, California) was used to carry let-7g cDNA (LV-let7g) or let-7g sponge (LV-let7g-sponge) as described previously [13]. LV-let7g (1 × 10^7^ TU diluted in 1% w/v body weight PBS, typically 0.2–0.25 ml) was injected weekly via the tail vein for 12 weeks, while LV-let7g-sponge was injected weekly for 6 weeks or 9 weeks. Lentivirus carrying the empty vectors was used as a negative control.

After the completion of the treatment, mice were killed by an overdose of isofluorane anesthesia, and were perfused with PBS instillation, followed by constant-pressure infusion of 10% neutral buffered formalin for 30 min. After the removal of the blood, the aortas were dissected, opened longitudinally and mounted on glass slides for oil red staining on the entire aorta to assess the severity of atherosclerosis. Images of the aortas were captured with a digital camera utilizing a microscope. Data were reported as the percentage of the aortic surface covered by atherosclerotic lesions.

For some animals, the aortic roots were removed, and embedded in Sakura Tissue-Tek® O.C.T compound. Serial sections (8 μm) of the aortic root were cut using a Thermo Scientific Shandon Cryostat FSE, and sections were immunofluorescence stained using a MAC3 antibody for macrophages (1/100 dilution, BD Biosciences), anti-α-actin (1/100 dilution, Millipore) and IKKα (1/100 dilution, Genetex). MAC3 was visualized with a DyLight™ 649-conjugated anti-rat IgG antibody (H&L). α-actin and IKKα were visualized with a DyLight™ 488-conjugated anti-rabbit IgG antibody (H&L), respectively. Cell nuclei were labeled with 0.1 mg/ml DAPI (Molecular Probes, Carlsbad, CA). Apoptotic cells in atherosclerotic lesions were detected by the TUNEL assay.

### Target gene prediction

Three algorithms were used to predict let-7g target genes. The algorithms are miRanda algorithm (http://microrna.sanger.ac.uk/targets/v5/), TargetScan (http://targetscan.org/) and PicTar (http://pictar.mdc-berlin.de/).

### MicroRNA and siRNA transfection

The sequences of let-7g mimic and negative control miRNA (NC-miR) (Life Technologies) are: let-7g mimic, 5′- UGAGGUAGUAGUUUGUACAGUU -3′; negative control sequence, 5′-AGUACUGCUUACGAUACGG-3′. The NC-miR has been extensively tested and validated to produce no identifiable effects on known miRNA function in human cells or tissues. By using Lipofectamine 2000 reagent (Sigma) or HiPerFect transfection reagent (Qiagen), let-7g mimic or NC-miR was transfected into THP-1 derived macrophages for 24 hours. For siRNA transfection, we used the following products: universal negative control siRNA#1 (Sic001, Sigma-Aldrich), RelA siRNA (SASI_Hs01_00171091, Sigma-Aldrich), p105 siRNA (SASI_Hs01_00227679, Sigma-Aldrich), RelB siRNA (SASI_Hs01_00103187, Sigma-Aldrich), p100 siRNA (SASI_Hs01_00138464, Sigma-Aldrich).

### Western blot for protein levels

THP-1 derived macrophages were homogenized in 100 μl of protein extraction reagent (Thermo Scientific) and protease inhibitor (Panomics). Protein concentration was determined by Pierce BCA Protein Assay Kit (Thermo Scientific). 20 μg protein was loaded per lane and separated by NuPAGE Novex Bis-Tris 4–12 % mini gel electrophoresis (Invitrogen) in the Novex Xcell-II apparatus for 120 min at 100 V, and transferred to Immbilon-PVDF transfer membranes (Millipore) for immunoblotting. Proteins were visualized by enhanced chemiluminescence according to the manufacturer's instruction. Nonspecific binding was blocked with 5% nonfat milk for 1 h at the room temperature. The antibodies to IKKα, IKKβ, IKKγ, IκBα, p-IκBα and β-actin were purchased from Genetex. The antibodies to phosphorylated-IKKα (phospho Ser176/Ser180), phosphorylated IKKβ (phospho Ser177/Ser181), phosphorylated IKKγ (Ser85) were purchased from Biocompare. The antibody to IκBβ was purchased from Abcam. The antibody to p-IκBβ was purchased from Cell signaling. The antibodies to Bcl-2 and FasL were purchased from BD Biosciences (Pharmingen). The antibodies to p50, p52, Rel A, Rel B and Lamin B were purchased from Santa Cruz. The antibody to p-53 was purchased from Sigma. The antibody to Noxa was purchased from Calbiocam. The antibodies to ABCA1, PUMA, Bax, activated caspase3 and GAPDH were purchased from Millipore.

### Cell nuclear extract

Cell nuclear extracts were prepared by the NucBuster™ Protein Extraction kit (Novagen). The protein concentrations of nuclear extracts were determined by the Pierce BCA Protein Assay Kit (Thermo Scientific) using BSA as a standard.

### Detection of DNA fragmentation by the TUNEL assay

The ApoAlert DNA fragmentation assay kit (Clontech) was used for the TUNEL assay. The incorporation of fluorescein-dUTP into the fragmented nuclear DNA generates the green fluorescence detected by a standard fluorescein filter set (520 ± 20 nm). All cells stained with propidium iodide (PI) exhibited strong red cytoplasmic fluorescence when viewed at > 620 nm.

### Subcellular localization of NF-κB proteins by confocal microscopy

THP-1 derived macrophages were seeded onto Millicell EZ slide (Millipore) 4 well glass slide and infected with LV-control vector, LV-let7g or LV-let7g-sponge. The macrophage monolayers were washed with PBS and fixed for 10 min in PBS containing 4% paraformaldehyde, permeabilized with PBS containing 0.1% Triton X-100 and then blocked with 5% BSA in PBS. Subsequently, cells were incubated with anti-bodies (1:100) against RelA, p50, RelB or p52 for 2 hr at room temperature, followed by incubation with the secondary anti-bodies conjugated with DyLight^TM^ 488-conjugated anti-rabbit or anti-Mouse IgG (H&L) for 1 hr at room temperature in the dark. Macrophage nuclei were labeled with 0.1 mg/ml DAPI (Molecular Probes, Carlsbad, CA) in PBS for 5 min at room temperature. Finally, the immunostained cells were mounted with PermaFluor (Thermo Scientific) on glass slides and the co-localization of RelA, p50, RelB and p52 with the nucleus analyzed by confocal laser microscopy (FV1000 IX-81; Olympus).

### IKKα 3′-UTR luciferase reporter construct and assay

IKKα 3′-UTR plasmid constructs were created to experimentally confirm the binding of let-7g to IKKα. A 236-bp segment of PCR product from the wild-type 3′-UTR containing one let-7g binding site was cloned into the Mlu I/Hind III site of the pMIR-REPORT Luciferase vector (Life Technologies). The mutant 3′-UTR was also generated by site-directed mutagenesis based on the two-step PCR megaprimer method as described previously [31]. The sequence of the mutant 3′-UTR of let-7g contains 5′-TGAAGAATAAATTCATGGAGC-3′ (the six mutated nucleotides were underscored). A construct (either wild or mutant type) and let-7g mimic were co-transfected into the HEK293 cells, and firefly and Renilla luciferase activity were measured using the Dual-Luciferase Reporter Assay (Promega) at 24 hours after transfection.

### Chromatin immunoprecipitation (ChIP) assay

ChIP assays were performed according to the manufacturer's instructions (EZChIP; Millipore). Macrophages were fixed with 1% formaldehyde, washed with PBS, and scraped. After centrifugation, the cells were lysed in 300 μL of lysis buffer (50 mM Tris at pH 8.0, 5 mM EDTA, 1% SDS, and protease inhibitor mixture). The cell lysate was homogenized by sonication, diluted by 1.7 mL of dilution buffer (20 mM Tris at pH 8.0, 5 mM EDTA, 100 mM NaCl, 2 mM EDTA, 0.5% Triton X-100, and protease inhibitor mixture), and precleaned with salmon sperm DNA (ssDNA)-saturated protein A/G Sepharose. One-tenth of the volume of cell lysate was kept as the input. Immunoprecipitation was performed using antibodies against p50, Rel A, p52 and Rel B. The immune complexes were collected with ssDNA-saturated protein A/G Sepharose and washed with a low-salt buffer (20 mM Tris at pH 7.4, 0.1% SDS, 1% Triton X-100, 2 mM EDTA, 150 mM NaCl) followed by a high-salt buffer (20 mM Tris at pH 7.4, 0.1% SDS, 1% Triton X-100, 2 mM EDTA, 250 mM NaCl). The immunoprecipitated chromatin was eluted with an elution buffer (0.1 M NaHCO3, 1% SDS). Cross-link was reversed by incubating the input and eluted chromatin at 65°C overnight. After proteinase K treatment for 1 h at 55°C, DNA was purified by using the DNA purification kit (Qiagen) and analyzed by quantitative real-time PCR. The primers used in Chip assay are listed in Supplementary Table 1.

### ChIP-sequencing

Chromatin extracted from 1 × 10^7^ oxLDL-treated THP-1 macrophages was used for the p52-ChIP experiment. The ChIP-Seq DNA Sample Preparation Kit (Illumina, 1003473) was used to precipitate p52 and purify ChIP DNA (350 ng). Prior to sequencing, the DNA was quantified using a NanoDrop 1000 Spectrophotometer, and the quality of DNA was assessed using a Bioanalyzer DNA 1000 (Agilent). The ChIP DNA ends were repaired using Klenow enzyme, T4 DNA polymerase, and T4 PNK, followed by treatment with Klenow fragment to generate a protruding 3′A base used for adaptor ligation. Following ligation of a pair of Illumina Truseq DNA adapters to the repaired ends, the ChIP DNA was amplified and the fragments around 250–400 bp were isolated from agarose gel for sequencing. The enriched genomic regions from the ChIP-seq experiments were determined using CisGenome software [32] by two-sample analysis to identify enriched regions. CisGenome two sample analysis determined the regions where the ChIP signals were enriched relative to the control ChIP (IgG) using a conditional binomial model that involves 100-bp windows passing a 0.1 FDR cut-off.

### RNA-Sequencing (RNA-Seq) experiment

The RNA-sequencing library was prepared from approximately 4 mg total RNA of THP-1 macrophage transfected with RelA-siRNA and p105-siRNA to block the canonical pathway (Supplementary Table 4) or control siRNA using Illumina TruSeq RNA sample Preparation kit (Illumina, Inc., San Diego, CA). Parallel sequencing was performed using an Illumina Genome Analyzer II (Illumina, Inc., San Diego, CA). Single-end sequence reads between 30 and 51 bp were generated, and RNA-seq read quality was evaluated based on the Illumina purity filter, percent low quality reads, and distribution of phred-like scores at each cycle. Reads were aligned to the human genome (hg19) with TopHat 2.0.5 (http://tophat.cbcb.umd.edu/), using the Ensembl 63 GTF file for gene models. Transcript abundance and gene expression levels were quantitated using Cufflinks 2.0.1 (http://cufflinks.cbcb.umd.edu/). Expression values were normalized using fragments per kilobase of exon per million mapped reads (FPKM). Gene Ontology (GO) was carried out by using DAVID (http://david.abcc.ncifcrf.gov/).

### Statistical analysis

Statistical differences were evaluated by Student's *t*-test. A *p* value less than 0.05 is considered statistically significant in all experiments. Analysis of the data and plotting of the figures were performed by the SigmaPlot 10 software (Systat Software Inc., CA, USA).

## SUPPLEMENTARY MATERIALS FIGURES AND TABLES


