# Supplementary material for: Let-7g suppresses both canonical and non-canonical NF-κB pathways in macrophages leading to anti-atherosclerosis
Source: Oncotarget. 2017 May 23;8(60):101026–41. doi: 10.18632/oncotarget.18197 (PMC5731853; doi:10.18632/oncotarget.18197)
Supplement: Supplementary file 1 [file oncotarget-08-101026-s001.pdf]

Let-7g suppresses both canonical and non-canonical NF-κB pathways in macrophages leading to anti-atherosclerosis

SUPPLEMENTARY MATERIALS

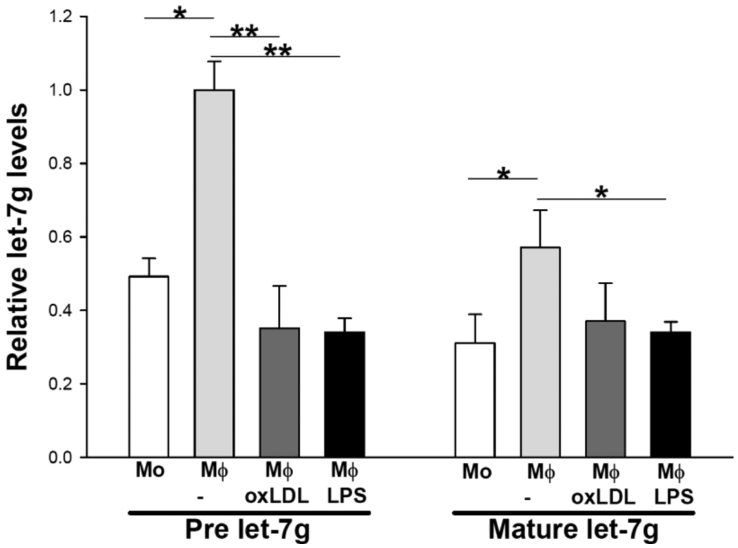

Supplementary Figure 1: Quantitative analysis of northern blot for biotin labeled mature and pre-microRNA let-7g in monocyte or macrophage treated with oxLDL or LPS. The amount of RNA loaded in each well was normalized to the amount of U6. Data are presented as mean ± SEM. $^{*}< 0.05$; $^{**}< 0.01$.

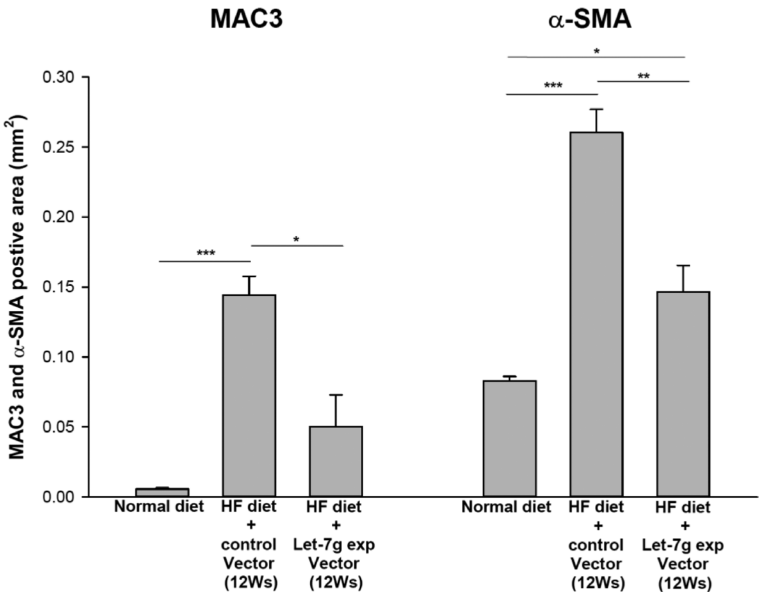

Supplementary Figure 2: Quantitative analysis of fluorescently labeled MAC3 and α-SMA of let-7g's effect in apoE KO mice under a HF diet for 12 weeks. Data are presented as mean ± SEM from three independent experiments. $^{*}< 0.05$; $^{**}< 0.01$; $^{***}< 0.001$.

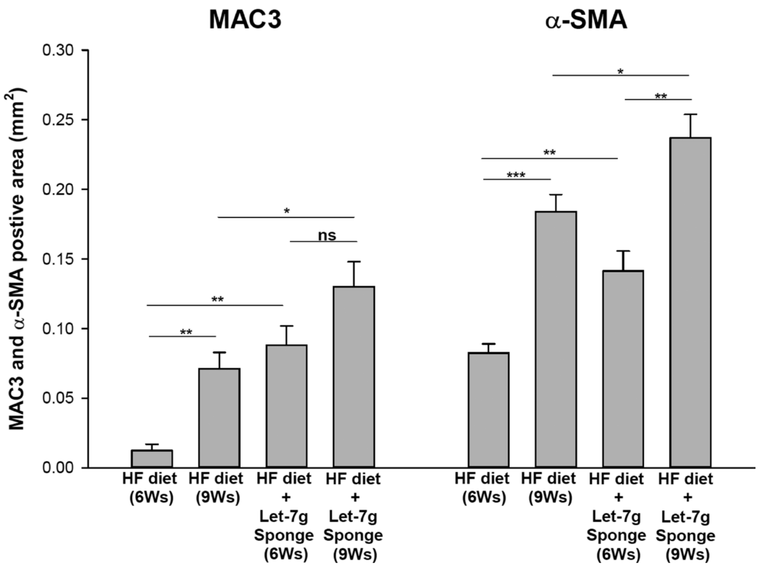

Supplementary Figure 3: Quantitative analysis of fluorescence labeled MAC3 and α-SMA of let-7g's knock-out effect in apoE KO mice under a HF diet for 6 or 9 weeks. Data are presented as mean ± SEM from three independent experiments. *< 0.05; **< 0.01; ***< 0.001.

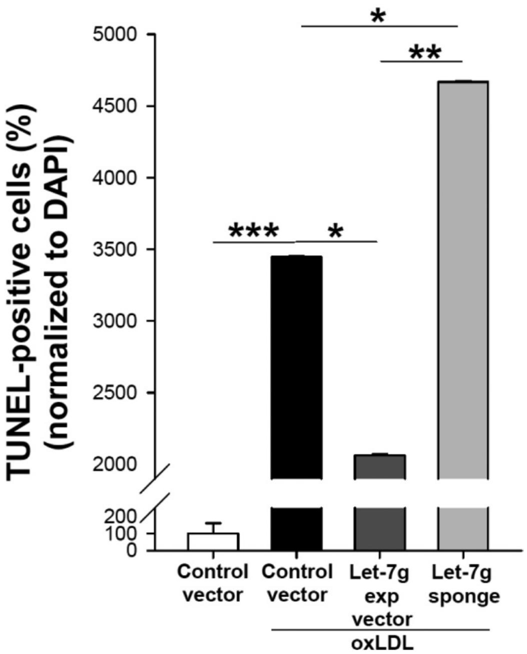

Supplementary Figure 4: Quantitative analysis of TUNEL-positive macrophages of let-7g's effect. Data are presented as mean ± SEM from three independent experiments. *< 0.05; **< 0.01; ***< 0.001.

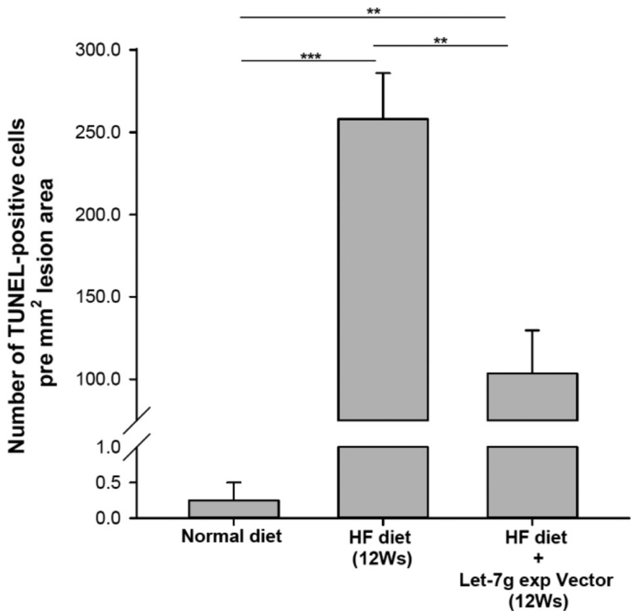

Supplementary Figure 5: Quantitative analysis of TUNEL-positive cells in apoE KO mice aortic sections of let-7g's effect. Data are presented as mean ± SEM from three independent experiments. $^{*}< 0.05$; $^{**}< 0.01$; $^{***}< 0.001$.

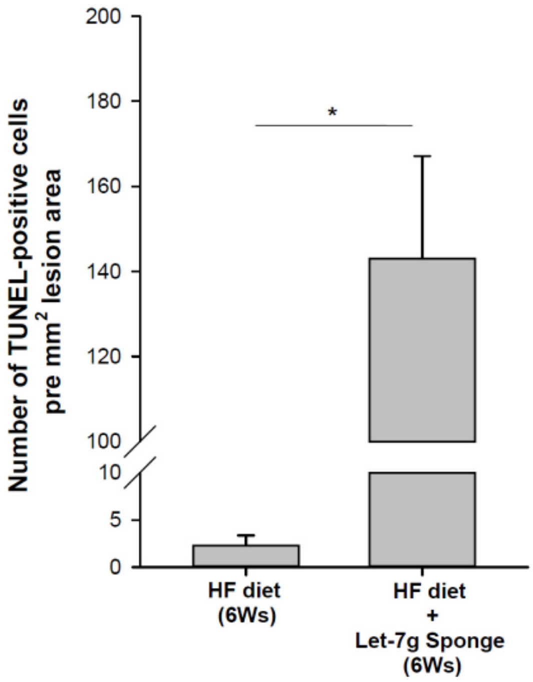

Supplementary Figure 6: Quantitative analysis of TUNEL-positive cells in apoE KO mice aortic sections of let-7g's knock-out effect. Data are presented as mean ± SEM from three independent experiments. $^{*}< 0.05$; $^{**}< 0.01$; $^{***}< 0.001$.

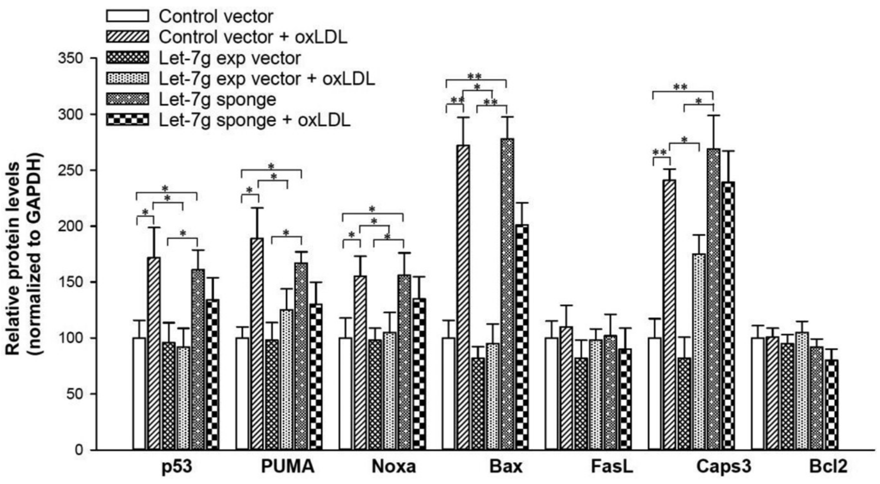

Supplementary Figure 7: Semi-quantitative western blot data of let-7g's effect on the p53-signaling in oxLDL-treated macrophages.

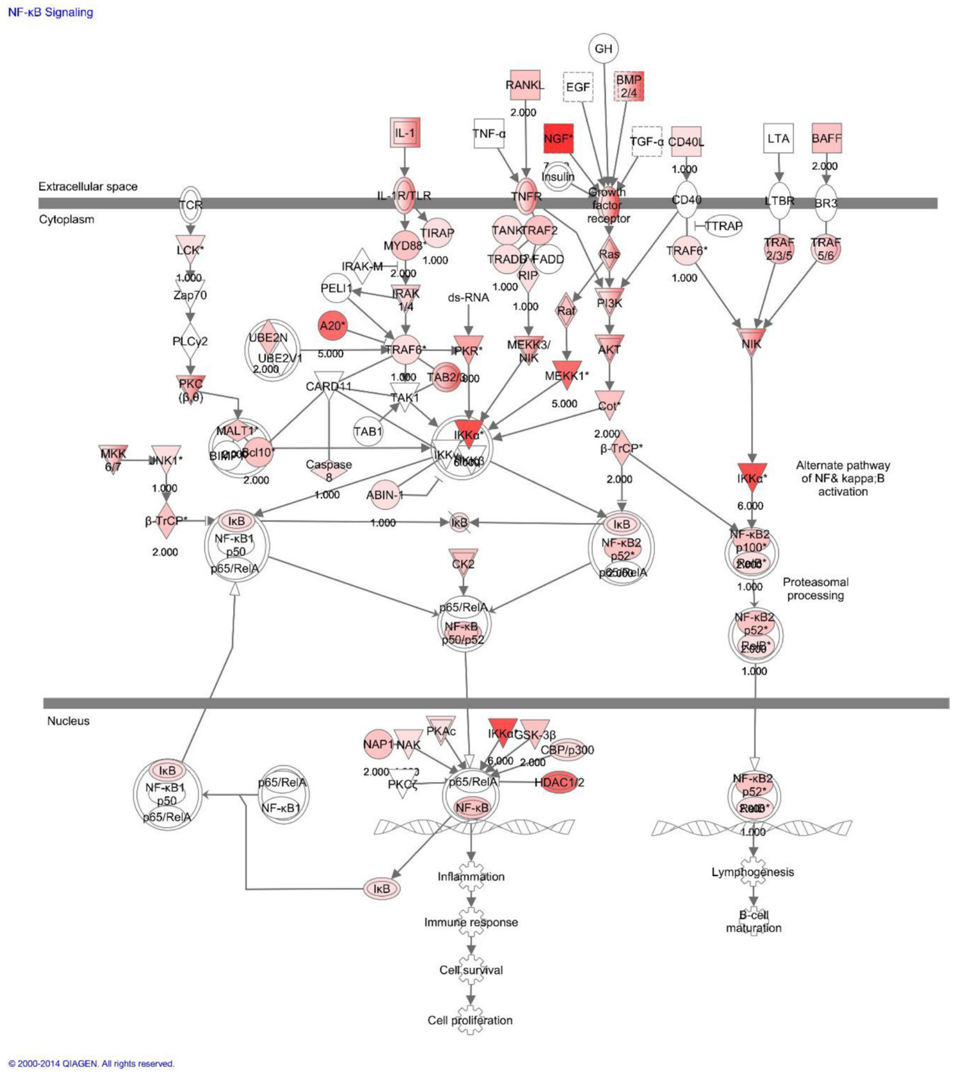

Supplementary Figure 8: Potential let-7g target genes involved in the NF-κB signaling pathway by ingenuity pathway analysis (IPA).

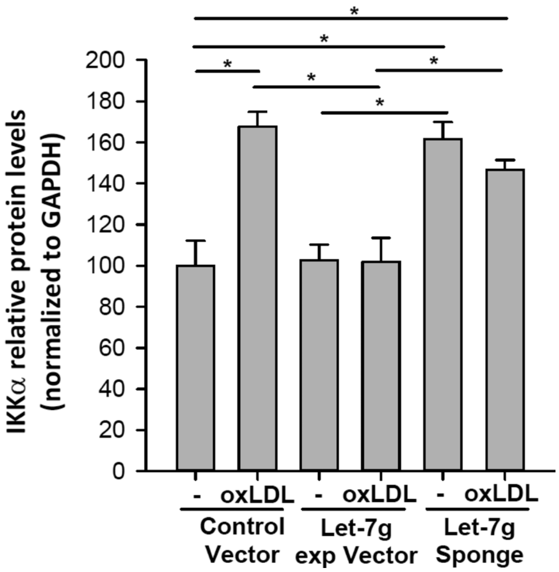

Supplementary Figure 9: Semi-quantitative western blot data of let-7g's effect on IKKα relative proteins expression in macrophages. Data are presented as mean ± SE from 3 independent experiments. *< 0.05; **< 0.01; ***< 0.001.

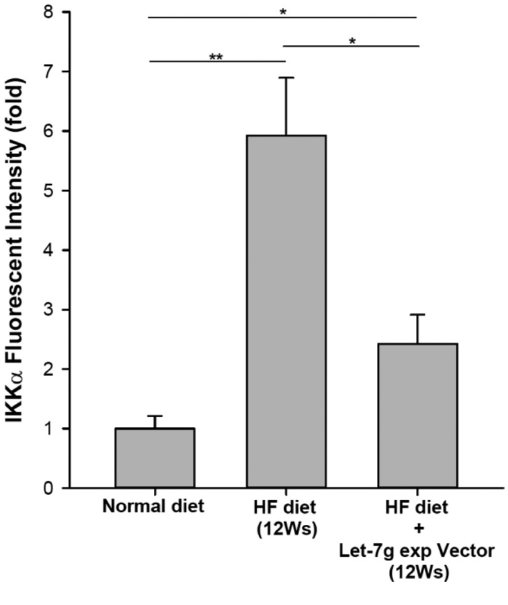

Supplementary Figure 10: Quantitative analysis of fluorescence labeled plaques of let-7g's effect on IKKα expression in apoE KO mice under a HF diet for 12 weeks. Data are presented as mean ± SEM from three independent experiments. *< 0.05; **< 0.01.

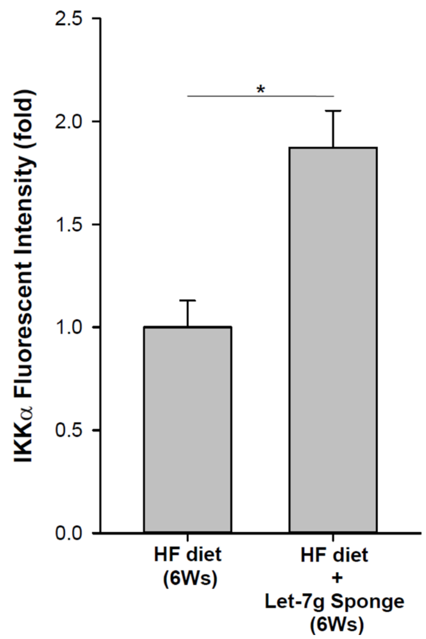

Supplementary Figure 11: Quantitative analysis of fluorescence labeled plaques of let-7g's knock-out effect on IKKα expression in apoE KO mice under a HF diet for 6 weeks. Data are presented as mean ± SEM from three independent experiments. *< 0.05

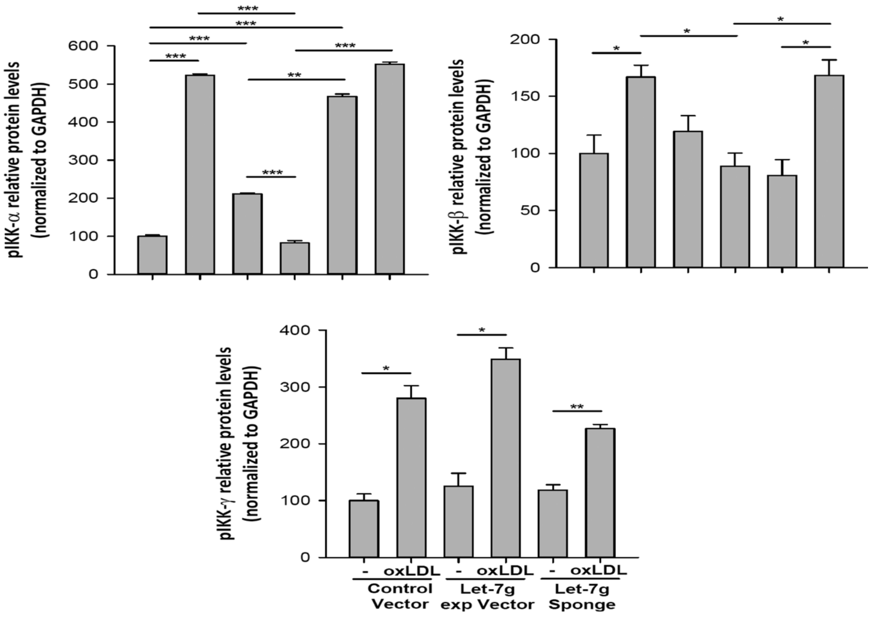

Supplementary Figure 12: Semi-quantitative western blot data of let-7g's effect on phosphorylated-IKKα, IKKβ and IKKγ relative proteins expression in macrophages. Data are presented as mean ± SE from 3 independent experiments. *<0.05; **< 0.01; ***< 0.001.

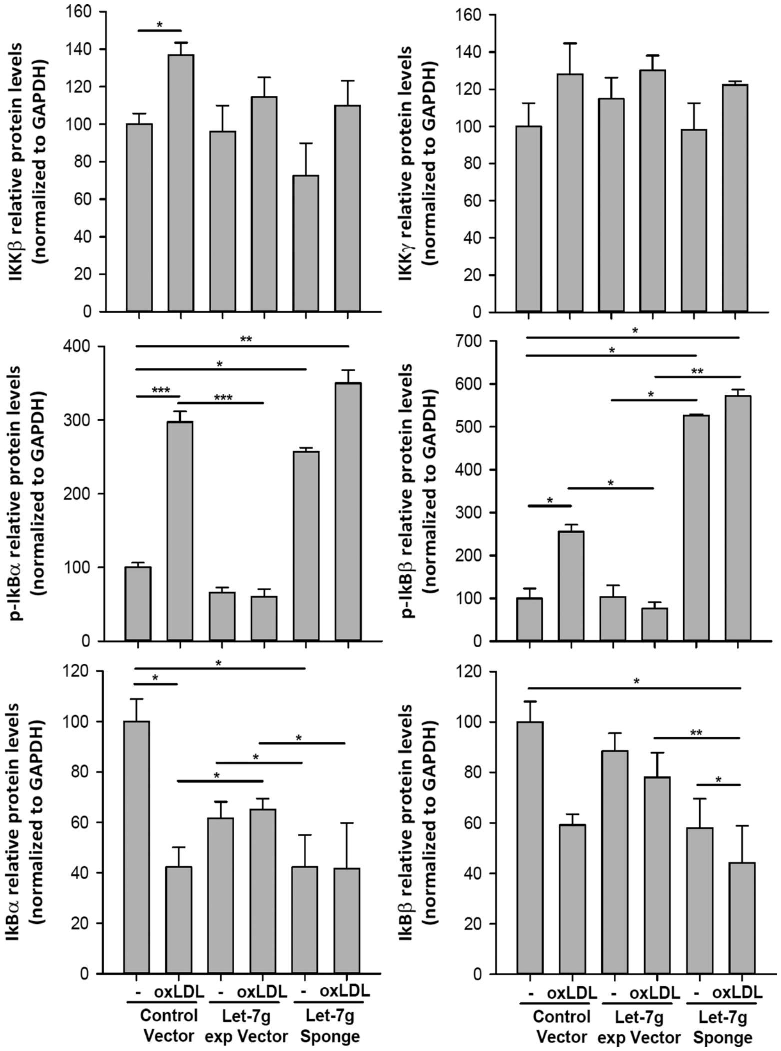

Supplementary Figure 13: Semi-quantitative western blot data of let-7g's effect on IKKβ, IKKγ, IκBα, IκBβ, phosphorylated-IκBα (p-IκBα) and p-IκBβ relative proteins expression in macrophages. Data are presented as mean ± SEM from 3 independent experiments. *< 0.05; **< 0.01; ***< 0.001.

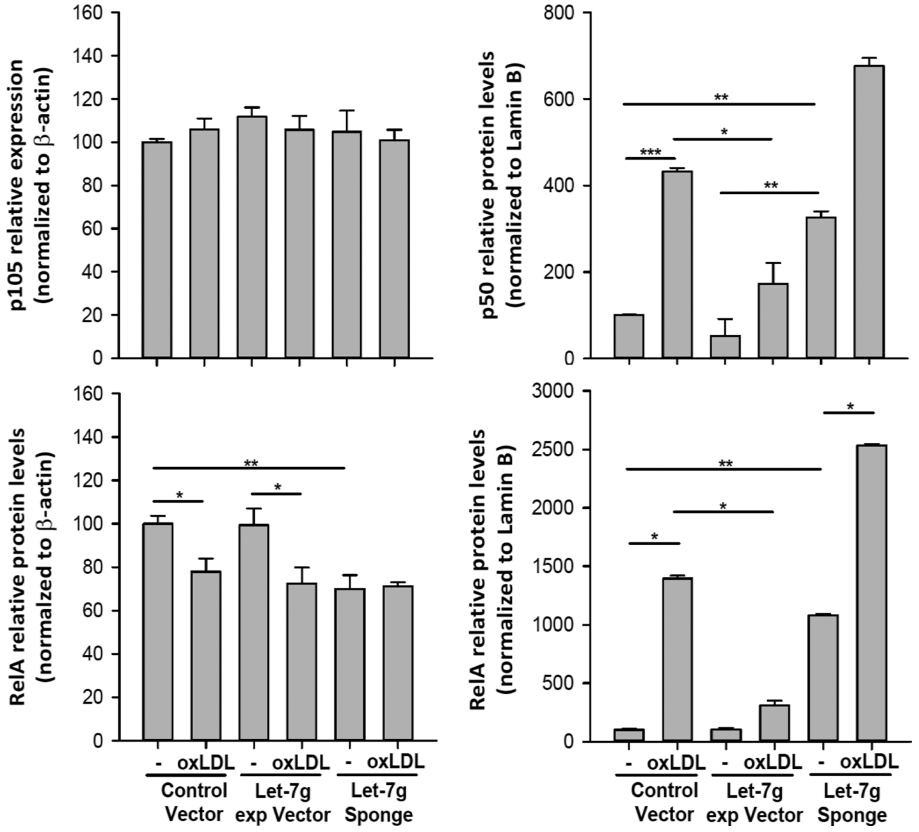

Supplementary Figure 14: Semi-quantitative western blot data of let-7g's effect on p105, p50 and RelA relative proteins expression in macrophages. Data are presented as mean ± SEM from 3 independent experiments. $^{*}< 0.05$; $^{**}< 0.01$; $^{***}< 0.001$.

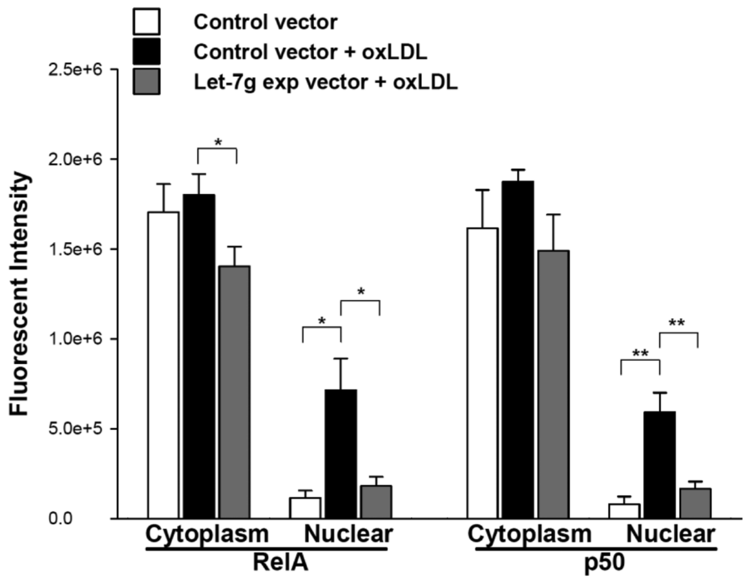

Supplementary Figure 15: Semi-quantitative analysis of RelA or p50 fluorescence in the oxLDL treated-macrophages treated with control vector or Let-7g exp vector. Data are presented as mean ± SEM from 3 independent experiments. $^{*}< 0.05$; $^{**}<$ 0.01

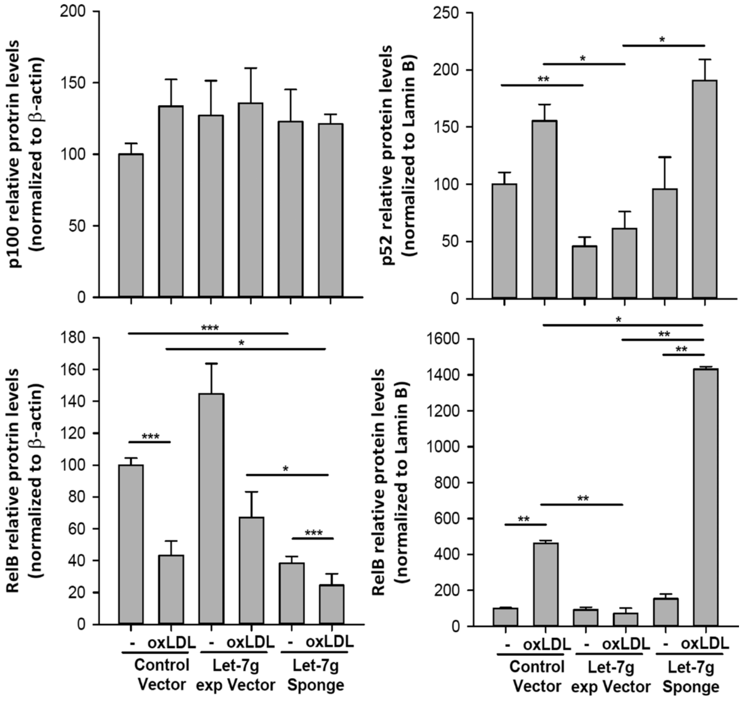

Supplementary Figure 16: Semi-quantitative western blot data of let-7g's effect on p100, p52 and RelB relative proteins expression in macrophages. Data are presented as mean ± SEM from 3 independent experiments. *< 0.05; **< 0.01; ***< 0.001.

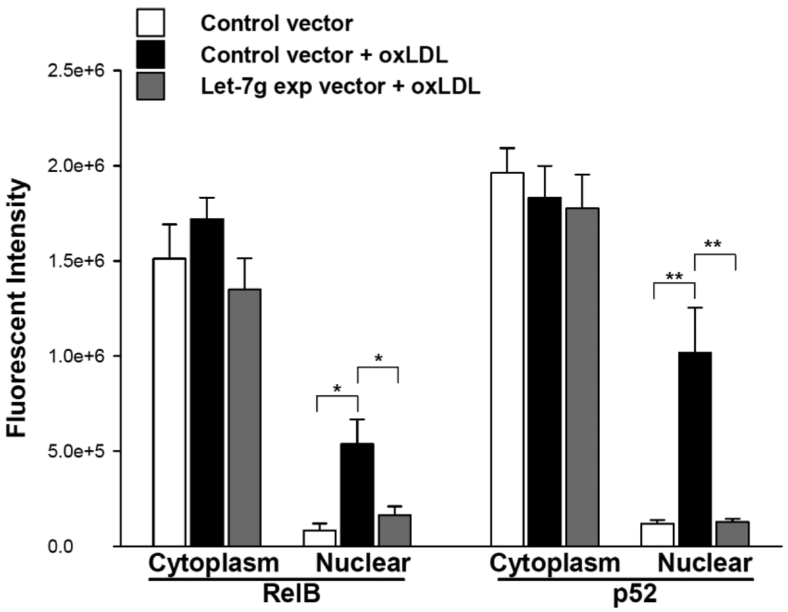

Supplementary Figure 17: Semi-quantitative analysis of RelB or p52 fluorescence in the oxLDL traeted-macrophages treated with control vector or Let-7g exp vector. Data are presented as mean ± SEM from 3 independent experiments. *< 0.05; **< 0.01.

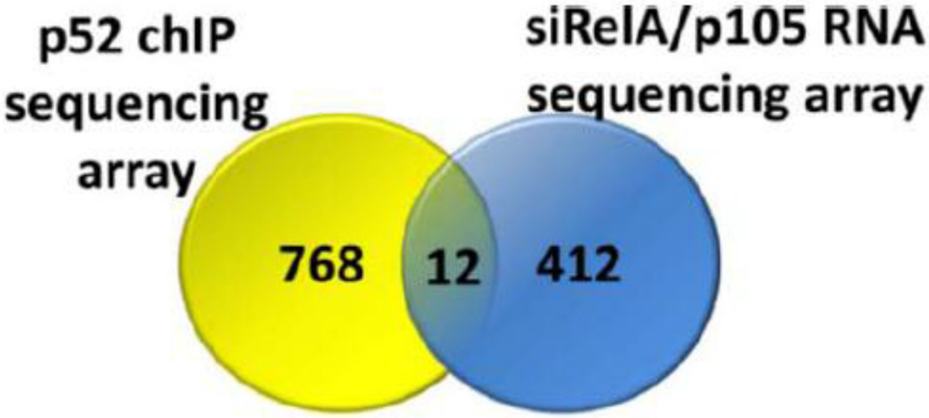

Supplementary Figure 18: Venn diagram for Chip-seq and RNA-seq experiments. Venn diagram shows p52 binding sites identified by p52 ChIP-seq and RNA-seq experiments in macrophages co-transfected with RelA-siRNA and p105-siRNA during foam cell formation. Twelve p52-affected and differentially expressed transcripts in the non-canonical pathway are indicated by the combination of results from ChiP-seq and RNA-seq experiments.

Supplementary Table 1: Primer sets

Method	Gene	Primer Sequence	Reference
QRT-PCR	**IKKα**	F: 5′-CAGCCATTTACCTGGCATGAG-3′<br>R: 5′-GAGGGTCCCAATTCAACATCAA-3′	[1]
	**p53**	F: 5′-CCCAAGCAATGGATGATTTGA-3′<br>R: 5′-GGCATTCTGGGAGCTTCATCT-3′	[2]
	**GAPDH**	F: 5′-ACCCACTCCTCCACCTTTGA-3′<br>R: 5′-CTGTTGCTGTAGCCAAATTCGT-3′	[3]
	**SREBF2**	F: 5′-CGAATTGAAAGACCTGGTCATG-3′<br>R: 5′-TCCTCAGAACGCCAGACTTGT-3′	[4]
ChIP Assay	**SREBF2**		
	(-1615 bp region)	F: 5′-CAACTCCTGACCTCAAATGATCTG-3′<br>R: 5′-GCTCTCGGCATAAGCTTTGG-3′	
	(-15 bp region)	F: 5′-CCCATTGACAACAAACAG-3′<br>R: 5′-CCGCCATGTTTGCGTTGC-3′	
Genotyping	**Cre**		[5]
	NLSCre	5′-CCCAAGAAGAAGAGGAAGGTGTCC-3′	
	Cre8	5′-CCCAGAAATGCCAGATTACG-3′	
	**apoE**		[6]
	Primer 1	5′-GCCTAGCCGAGGGAGAGCCG-3′	
	Primer 2	5′-TGTGACTTGGGAGCTCTGCAGC-3′	
	Primer 3	5′-GCCGCCCCGACTGCATCT-3′	

Supplementary Table 2: 29 NF-κB related genes

let7g and NFkB	DIANAmT	miRanda	miRDB	miRWalk	RNAhybrid	PICTAR5	PITA	RNA22	Targetscan	SUM
AZI2	0	1	0	1	0	0	0	0	0	2
BCL10	0	1	0	1	0	0	0	0	0	2
BTRC	0	1	0	1	0	0	0	0	0	2
CASP8	0	0	0	0	0	1	0	0	0	1
CD40LG	0	0	0	0	0	1	0	0	0	1
IKKα	1	1	1	1	0	1	0	0	1	6
EIF2AK2	1	0	0	0	0	1	1	0	0	3
GSK3B	0	1	0	1	0	0	0	0	0	2
LCK	0	1	0	0	0	0	0	0	0	1
MALT1	0	0	0	0	0	1	1	0	0	2
MEKK1	1	1	0	1	0	1	0	0	1	5
MAP3K8	0	1	0	1	0	0	0	0	0	2
MAPK8	1	0	0	0	0	0	0	0	0	1
MYD88	0	1	0	1	0	0	0	0	0	2
NFKB2	0	1	0	1	0	0	0	0	0	2
NGF	1	1	1	1	0	1	0	1	1	7
RELB	0	1	0	0	0	0	0	0	0	1
RIPK1	0	0	0	0	0	1	0	0	0	1
TANK	0	1	0	0	0	0	0	0	0	1
TBK1	0	1	0	0	0	0	0	0	0	1
TIRAP	0	0	0	0	0	1	0	0	0	1
A20	1	1	0	1	0	1	0	0	1	5
TNFSF11	0	1	0	1	0	0	0	0	0	2
TNFSF13B	0	1	0	0	0	1	0	0	0	2
TNIP1	0	1	0	0	0	0	0	0	0	1
TRADD	0	0	0	0	0	1	0	0	0	1
TRAF2	0	1	0	1	0	0	0	0	0	2
TRAF6	0	0	0	0	0	1	0	0	0	1
UBE2N	0	1	0	1	0	0	0	0	0	2

Supplementary Table 3: Gene ontology annotation of 12 genes which were directly affected by p52 in the non-canonical pathway

	Fold Change	Molecule Function	Biological Process	Cellular Component
**Foam Cell Formation**				
**ATGL**	**2.21**	Protein binding	Lipid catabolic process	Cytosol
**MARCO**	**0.33**	Scavenger receptor activity	Receptor-mediated endocytosis	Membrane
**Macrophage Movement**				
**NPAS3**	**35.83**	Signal transducer activity	Locomotory behavior	Cytoplasm
**TNC**	**5.65**	Fibronectin binding	Extracellular matrix organization	Extracellular matrix
**DNAH11**		Microtubule motor activity	Microtubule-based movement	Axonemal dynein complex
**Response to Oxidative Stress**				
**RRM2B**	**2.33**	Oxidoreductase activity	Response to oxidative stress	Mitochondrion
**Cell Apoptosis**				
**DAB2IP**	**18.81**	Protein binding	Induction of apoptosis via death domain receptors	Cytoplasm
**OBSCN**	**0.19**	Protein binding	Positive regulation of apoptotic process	Cytosol
**Others**				
**THAP8**	**3.06**	DNA binding	NA	NA
**ZNF625**	**2.26**	DNA binding	Regulation of transcription	Nucleus
**ATAD3A**	**0.35**	ATP binding	Cell growth	Mitochondrion
**NUBPL**	**0.32**	ATP binding	Mitochondrial respiratory chain complex I assembly	Mitochondrion

Supplementary Table 4: List of siRNAs used in this study

siRNA	Accession No.	Sequence
p105 siRNA-1	NM_003998	GGGUAUAGCUUCCCACACU
p105 siRNA-2		AGUGUGGGAAGCUAUACCC
Rel A siRNA-1	NM_021975	GGAAUCCAGUGUGUGAAGA
Rel A siRNA-2		UCUUCACACACUGGAUUCC
p100 siRNA-1	NM_001077493	CUGUCAAGAUCUGUAACUA
p100 siRNA-2		UAGUUACAGAUCUUGACAG
Rel B siRNA-1	NM_006509	GAGAUUGUCGAGCCCGUGA
Rel B siRNA-2		UCACGGGCUCGACAAUCUC
Control siRNA-1		GAUCAUACGUGCGAUCAGA
Control siRNA-2		UCUGAUCGCACGUAUGAUC

REFERENCES

1. Jiang R, Xia Y, Li J, Deng L, Zhao L, Shi J, Wang X, Sun B. High expression levels of IKKalpha and IKKbeta are necessary for the malignant properties of liver cancer. Int J Cancer. 2010; 126:1263–74.
2. Scherz-Shouval R, Weidberg H, Gonen C, Wilder S, Elazar Z, Oren M. p53-dependent regulation of autophagy protein LC3 supports cancer cell survival under prolonged starvation. Proc Natl Acad Sci USA. 2010; 107:18511–16.
3. Shema-Yaacoby E, Nikolov M, Haj-Yahya M, Siman P, Allemand E, Yamaguchi Y, Muchardt C, Urlaub H, Brik A, Oren M, Fischle W. Systematic identification of proteins binding to chromatin-embedded ubiquitylated H2B reveals recruitment of SWI/SNF to regulate transcription. Cell Reports. 2013; 4:601–08.
4. Rice SJ, Lai SC, Wood LW, Helsley KR, Runkle EA, Winslow MM, Mu D. MicroRNA-33a mediates the regulation of high mobility group AT-hook 2 gene (HMGA2) by thyroid transcription factor 1 (TTF-1/NKX2-1). J Biol Chem. 2013; 288:16348–60.
5. Clausen BE, Burkhardt C, Reith W, Renkawitz R, Förster I. Conditional gene targeting in macrophages and granulocytes using LysMcre mice. Transgenic Res. 1999; 8:265–77.
6. Clarke MC, Figg N, Maguire JJ, Davenport AP, Goddard M, Littlewood TD, Bennett MR. Apoptosis of vascular smooth muscle cells induces features of plaque vulnerability in atherosclerosis. Nat Med. 2006; 12:1075–80.
